# Meta-analysis of the prognostic value of p-4EBP1 in human malignancies

**DOI:** 10.18632/oncotarget.23031

**Published:** 2017-12-07

**Authors:** Tao Zhang, Jianrong Guo, Huili Li, Jiliang Wang

**Affiliations:** ^1^ Department of Gastrointestinal Surgery, Union Hospital, Tongji Medical College, Huazhong University of Science and Technology, Wuhan, 430022, Hubei, China

**Keywords:** p-4EBP1, prognosis, cancer, meta-analysis, malignancies

## Abstract

Phosphorylated 4E-binding protein 1 (p-4EBP1) is the inactivated form of 4EBP1, which is a downstream mediator in the mTOR signaling pathway and a vital factor in the synthesis of some oncogenic proteins. This meta-analysis was conducted to assess the predicative value of p-4EBP1 expression in human malignancies. The PubMed and Embase databases were carefully searched. Articles comparing the prognostic worthiness of different p-4EBP1 levels in human malignancies were collected for pooled analyses and methodologically appraised using the Newcastle-Ottawa Scale (NOS). A total of 39 retrospective cohorts with an overall sample size of 3,980 were selected. Patients with lower p-4EBP1 expression had better 3-year (*P* < 0.00001), 5-year (*P* < 0.00001), and 10-year (*P* = 0.03) overall survival and better 3-year (*P* < 0.0001) and 5-year (*P* = 0.0005) disease-free survival. Subgroup analyses confirmed the unfavorable prognosis associated with p-4EBP1 overexpression. These findings were further validated by sensitivity analyses. Harbord and Peters tests revealed no publication bias within the included studies. It thus appears higher expression of p-4EBP1 indicates a poor prognosis in human malignancies.

## INTRODUCTION

Eukaryotic translation initiation factor 4E binding protein 1 (4EBP1) is a family of translation-repressor proteins, which is one of the two main downstream effectors (4EBP1and S6K1) of mammalian target of rapamycin (mTOR) [1]. 4EBP1 inhibits protein synthesis by binding to eIF4E. Dephosphorylated active 4EBP1 binds to eukaryotic translation initiation factor 4E (eIF4E) and disrupts the formation of the cap-dependent translation initiation complex, comprising the eIF4A RNA helicase, the eIF4E mRNA cap-binding protein, and the eIF4G scaffolding protein, which is essential for protein synthesis and is associated with cancer development and progression [2]. When 4EBP1 is phosphorylated and deactivated by upstream signals at several sites (Thr37/46, Thr70, Ser65, etc.), eIF4E is released from 4EBP1 and initiates cap-dependent translation to promote the synthesis of various proteins, including oncogenic proteins [3].

4EBP1 is a tumor suppressor and its deactivation (phosphorylation) might directly contribute to the development of multiple cancers [4]. Research have shown that loss of 4EBP1 function (phosphorylation of 4EBP1) can induce epithelial-mesenchymal transformation, migration, and invasion by cancer cells [5]. Accordingly, the expression of p-4EBP1 in tumor cells might indicate their oncogenic potential. Furthermore, studies have shown that the expression of p-4EBP1 in tumor tissues is a predictive biomarker of malignant potential.

However, despite the accumulating of evidence in the literature, the prognostic value of p-4EBP1 remains controversial. Benavente et al. [6] showed that a high level of p-4EBP1 correlates with a worse prognosis in patients with cervical cancer. Lee et al. [7] believed that gastric cancer patients with overexpression of p-4EBP1 had a prolonged survival time compared with low-expression patients. Azim et al. [8] reported that expression of p-4EBP1was not associated with prognosis in breast cancer patients.

To evaluate the prognosis and predictive value of the expression of p-4EBP1 in human malignancies, we conducted a comprehensive meta-analysis to provide theoretical support for clinical applications.

## MATERIALS AND METHODS

This meta-analysis was performed according to Cochrane Collaboration protocols and the PRISMA Checklist. Two investigators independently performed each step, and any disagreement was resolved by mutual discussion.

### Literature search

The Pubmed and Embase databases were searched by use of the terms (EIF4EBP1 protein OR Phosphorylated 4E-binding protein 1 OR p-4E-BP1 OR 4E-BP1 OR 4E-binding protein 1) AND (cancer OR carcinoma OR malignancy OR tumor). Abstracts and full-text of the preliminary articles were screened in sequence to ensure the eligibility of included articles.

### Selection criteria

The inclusion criteria were as follows: (1) English-language articles formally published until May 2017 and (2) studies that compare the prognostic value of different p-4EBP1 expressions in human malignancies. The elimination criteria were as follows: (1) Duplicated or overlapped articles, (2) review articles and case reports, and (3) articles with insufficient original data on survival analysis. Two investigators performed each evaluation independently to ensure the accuracy of the selection process.

### Data extraction

A standardized form was designed for the data-extraction process. Original data of elementary demographic characteristics (country, tumor type, TNM stage, detection method, p-4EBP1 level, phosphorylation site, subcellular localization, sample size, sex, age, and follow-up duration) and survival data (3-year overall survival, 5-year overall survival, 10-year overall survival, 3-year disease-free survival, 5-year disease-free survival, and 10-year disease-free survival) were extracted from text words, tables, or Kaplan-Meier curves. Survival data were mainly obtained from Kaplan-Meier curves and digitized by GetData Graph Digitizer 2.25 software.

### Methodological assessment

The Newcastle-Ottawa Scale (NOS) was utilized for methodological appraisal of the included observational studies. Three categories, including selection, comparability, and outcome, were established within the scale, with a maximum score of nine. Studies with scores of six or higher were graded as high quality .

### Statistical analysis

Statistical procedures were performed by Review Manager 5.3 software. The effect size was presented by odds ratio, and the Mantel-Haenszel model was utilized for dichotomous variables. The I^2^ value was adopted as a heterogeneity indicator among the included studies, and < 25%, < 50%, and > 50% implied low, moderate, and severe heterogeneity, respectively. A fixed-effects model was adopted for low heterogeneity, and a random-effects model was utilized for the remaining conditions. *P* < 0.05 indicated a statistical significance within the comparisons. Subgroup analyses were additionally conducted to explore potential confounding elements. The consistency of pooled outcomes was evaluated by sensitivity analysis. Publication bias was analyzed by the Harbord test and the Peters test.

## RESULTS

### General characteristics

**Figure 1:**
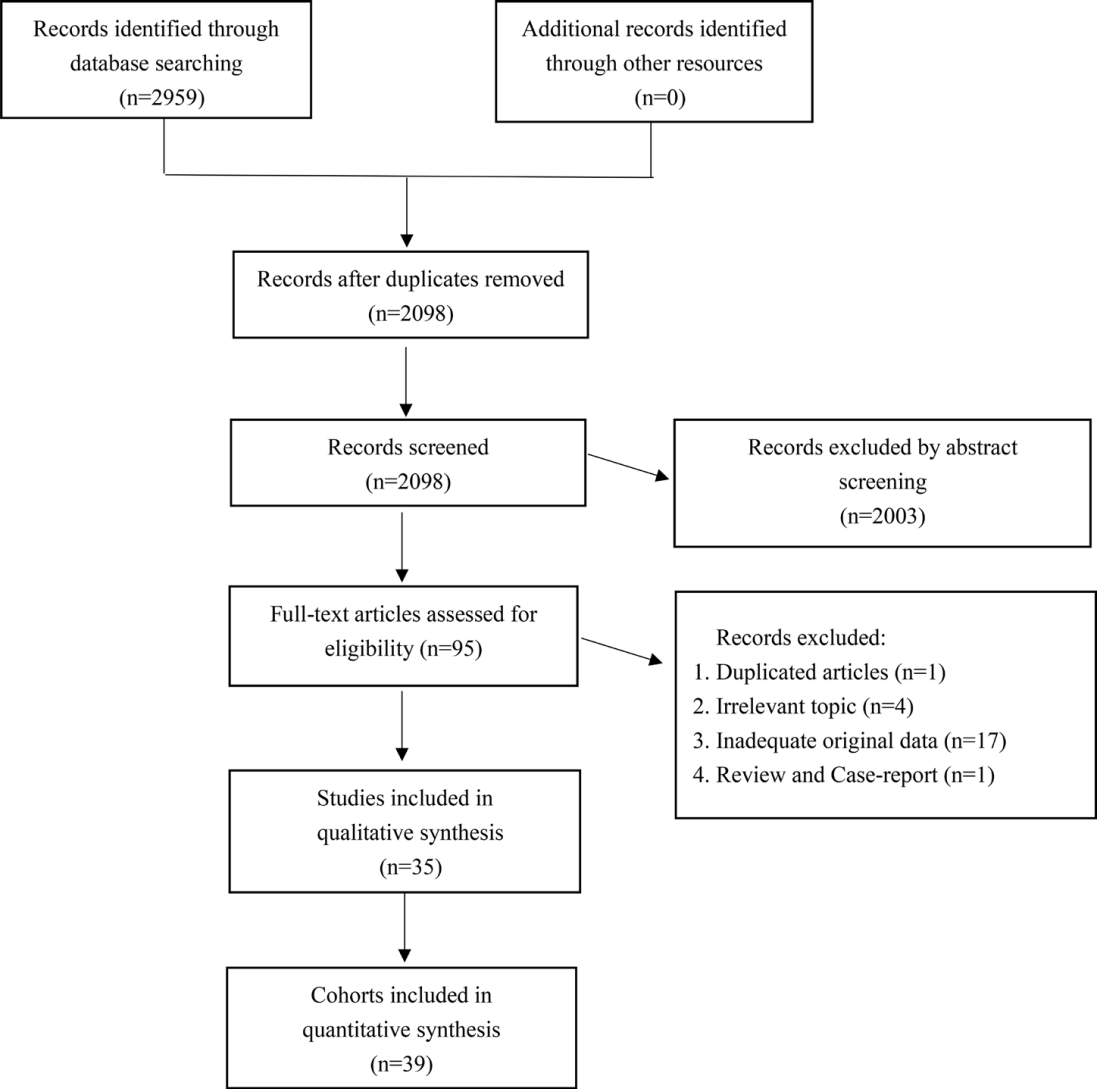
The selection flow chart of our meta-analysis

A total of 35 studies were selected from the initially retrieved 2,959 articles, which consisted of 39 retrospective cohorts (Figure 1). The sample size ranged from 30 to 285, with an overall sample size of 3,980 cases. The chief source country of the included investigations is China (*n* = 13), followed by Japan (*n* = 4), South Korea (*n* = 4), and Spain (*n* = 4); Greece (*n* = 3); Germany (*n* = 2) and the United States (*n* = 2); and France (*n* = 1), Italy (*n* = 1), and the UK (*n* = 1). Breast cancer (*n* = 6), lung cancer (*n* = 5), renal cancer (*n* = 3), and pancreatic cancer (*n* = 3) were the most frequent cancer types in the included articles. A total of 30 studies reported overall survival, and 14 studies reported disease-free survival. Other detailed baseline features were summarized and recorded in Supplementary Table 1 [6–40].

### Methodological assessment

Most of the included trials were graded as high quality in methodology, including 17 8-score studies, 10 7-score studies, and 13 6-score studies. Only Florio et al. [9] were evaluated as a low-quality study, with a 4-score on the Newcastle-Ottawa Scale (Supplementary Table 1).

### Prognostic significance of p-4EBP1 in survival analysis

**Figure 2:**
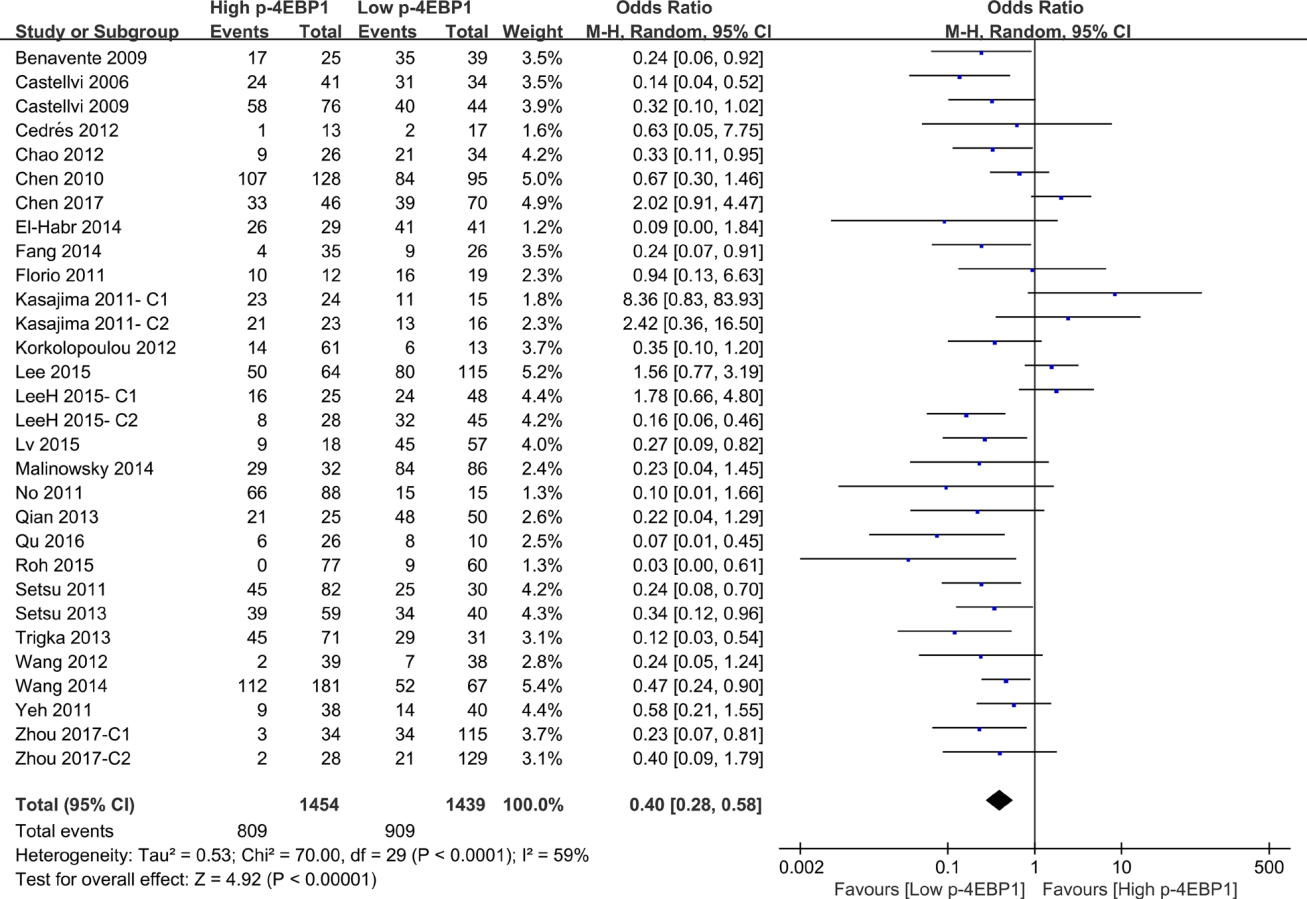
The correlation between p-4EBP1 expression and 3-year overall survival in malignancies

**Figure 3:**
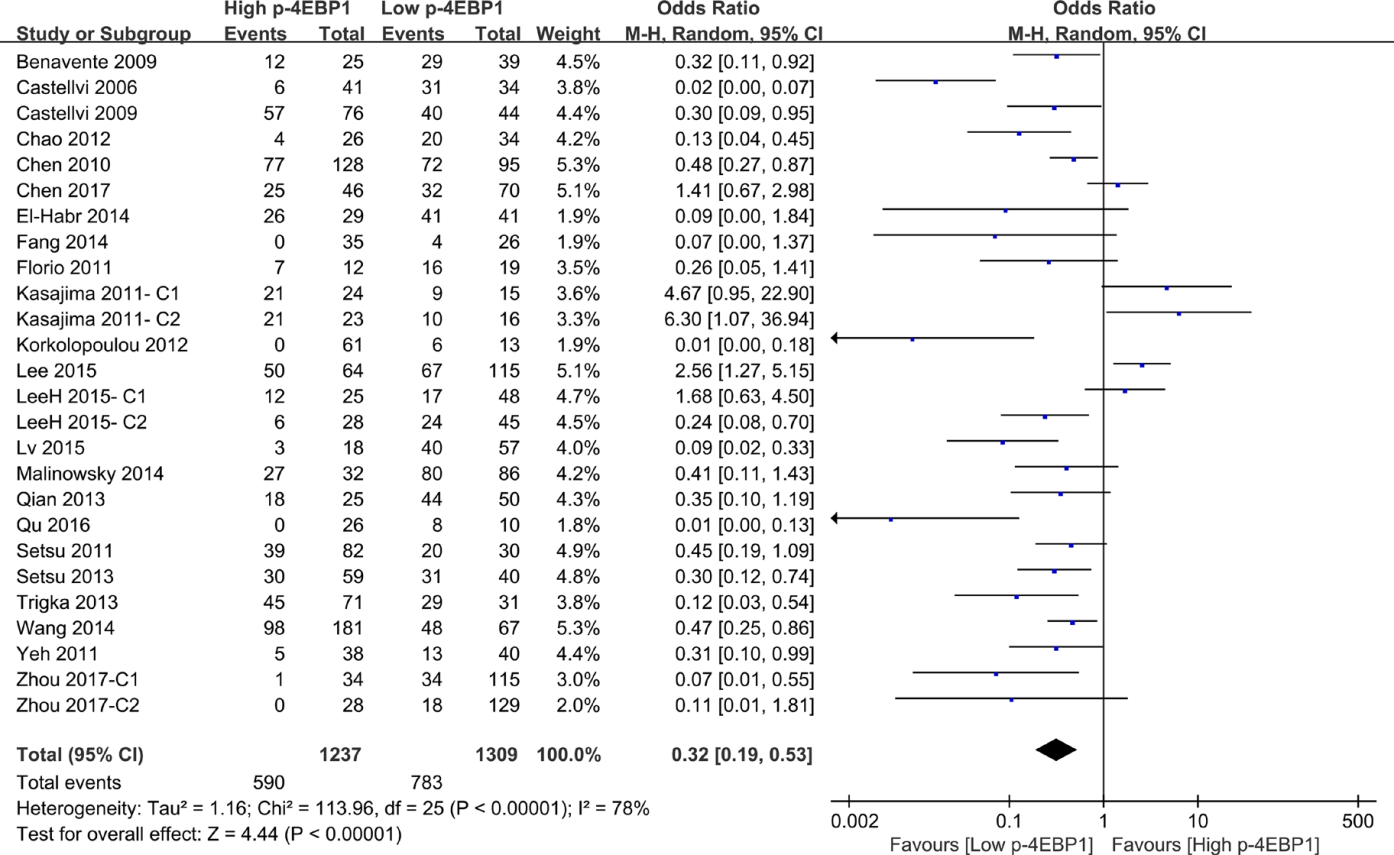
The correlation between p-4EBP1 expression and 5-year overall survival in malignancies

**Figure 4:**
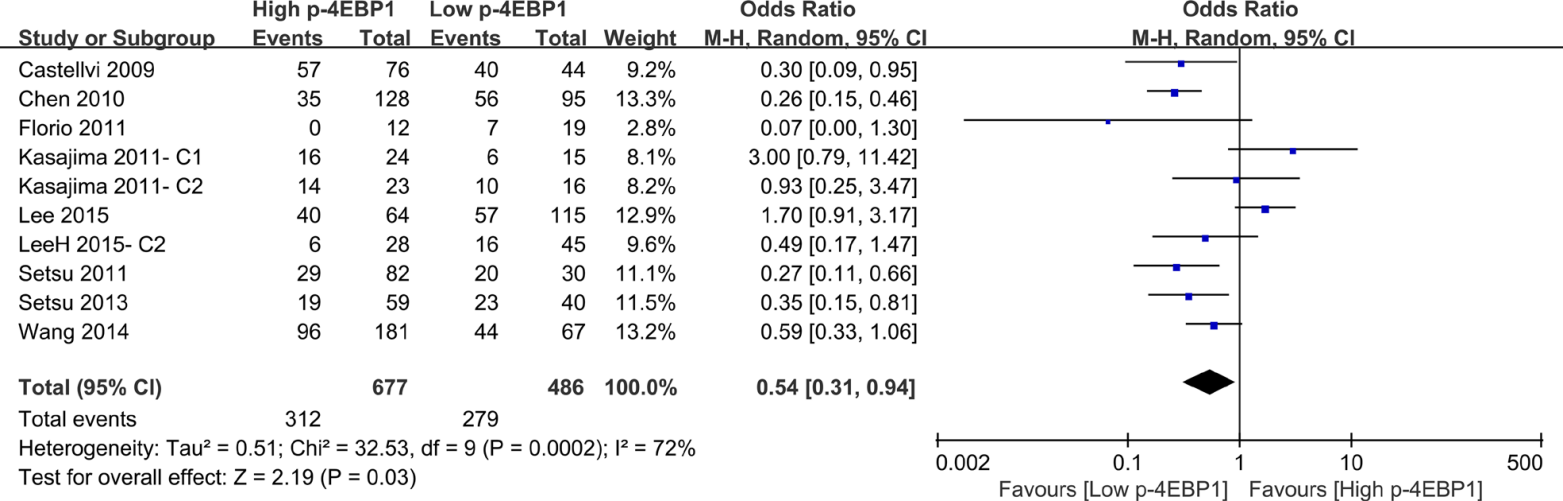
The correlation between p-4EBP1 expression and 10-year overall survival in malignancies

Compared with patients who had high p-4EBP1 expression, patients with lower p-4EBP1 had a better outcome for 3-year overall survival (*P* < 0.00001) (Figure 2). Our pooled analyses suggested that p-4EBP1 underexpression was a favorable indicator of 5-year overall survival among cancer patients, in contrast to those with redundant p-4EBP1 (*P* < 0.00001) (Figure 3). Furthermore, there was a correlation between patients with higher p-4EBP1 positivity and limited 10-year overall survival (*P* = 0.03) (Figure 4).

Patients with underexpressed p-4EBP1 had a superior outcome of 3-year and 5-year disease- free survival compared with patients who had a higher expression (*P* < 0.0001 and *P* = 0.0005). In contrast to patients with lower positivity, patients with p-4EBP1 overexpression obtained similar outcomes of 10-year disease-free survival (*P* = 0.64) (Supplementary Figure 1).

### Subgroup analyses

### Tumor type

Higher p-4EBP1 level indicated an unfavorable 3-year overall survival among patients with cholangiocarcinoma (*P* = 0.007), esophageal cancer (*P* = 0.03), lung cancer (*P* = 0.03), nasopharyngeal cancer (*P* = 0.02), ovarian cancer (*P* = 0.0009), pancreatic cancer (*P* = 0.02), and other types (*P* = 0.02). However, patients with colorectal cancer (*P* = 0.84) displayed similar prognosis regardless of different p-4EBP1 expressions (Supplementary Figure 2). Similarly, p-4EBP1 underexpression was a beneficial indicator of 5-year overall survival among patients with esophageal cancer (*P* = 0.0005), nasopharyngeal cancer (*P* = 0.0005), pancreatic cancer (*P* = 0.001), and other types (*P* = 0.01). Nevertheless, irrespective of colorectal cancer (*P* = 0.78) and lung cancer (*P* = 0.07), there was no significant difference in 5-year survival between the compared groups (Supplementary Figure 3).

Furthermore, p-4EBP1 isoform redundancy correlated to a worse 3-year disease-free survival in breast cancer (*P* = 0.003), renal cancer (*P* = 0.0004), and other types of cancer (*P* = 0.04). Additionally, underexpression of p-4EBP1 predicted a longer 5-year disease-free survival in breast cancer (*P* = 0.008) and renal cancer (*P* = 0.004) but not in other types of cancer (*P* = 0.15) (Supplementary Figure 4).

### Source region

All included studies were divided into Asian and non-Asian subgroups. Excessive p-4EBP1 expression was a negative indicator for 3-year (*P* = 0.0001 and *P* = 0.001) and 5-year (*P* = 0.0006 and *P* = 0.01) overall survival expectancy, both in Asian and in non-Asian subgroups (Supplementary Figures 5 and 6).

### Sex

Overexpression of p-4EBP1 indicated a worse 3-year and 5-year overall survival in the female-specific subgroup (*P* < 0.00001 and *P* = 0.003). Similar results were found in the non-sex-specific subgroup (*P* < 0.0001 and *P* = 0.0005) (Supplementary Figures 7 and 8).

### Mean age

p-4EBP1 isoform redundancy was significantly linked to unfavorable prognosis among patients with mean age < 60 years, irrespective of 3-year (*P* < 0.0001) and 5-year (*P* = 0.0003) follow-up duration. A significant correlation exists between p-4EBP1 overexpression and 3-year (*P* = 0.003) and 5-year (*P* = 0.02) overall survival among patients with mean age > 60 years (Supplementary Figures 9 and 10).

### TNM stage

Weaker p-4EBP1 positivity indicated longer 3-year and 5-year overall survival among patients with stage I–IV (*P* = 0.0005 and *P* = 0.001), stage I–II (*P* = 0.0002 and *P* = 0.002), stage II–IV (*P* = 0.01 and *P* = 0.003), and stage II–IV (*P* = 0.0008 and *P* = 0.003) cancers. However, patients with TNM stage IV had equivalent survival outcomes despite different p-4EBP1 expression (*P* = 0.71 and *P* = 0.48) (Supplementary Figures 11 and 12).

### Phosphorylation site

Underexpression of p-4EBP1 in phosphorylation sites threonine 37/46 (Thr37/46) (*P* = 0.0008) threonine 70 (Thr70) (*P* = 0.02) and serine 65 (Ser65) (*P* = 0.02) indicated prolonged 3-year overall survival among patients with malignancies. However, although high p-4EBP1 expression in phosphorylation sites Thr37/46 (*P* = 0.0006) and Ser65 (*P* = 0.0004) had a favorable outcome for 5-year overall survival, Thr70 had equivalent 5-year overall survival despite a different p-4EBP1 expression (*P* = 0.14) (Supplementary Figures 13 and 14).

### Subcellular localization

In subgroups characterized by different subcellular localization, our quantitative results suggested that trials based on cytoplasmic staining of p-4EBP1 correlated its over-reactivity with a worse 3-year (*P* = 0.03) overall survival but not with a worse 5-year overall survival (*P* = 0.15). There was no significant correlation between underexpression of nuclear p-4EBP1 and long-term prognosis among cancer patients (3-year overall survival: *P* = 0.27; 5-year overall survival: *P* = 0.25), although a significant correlation exists between overexpression of total p-4EBP1 and 3-year (*P* < 0.00001) and 5-year (*P* < 0.0001) overall survival (Supplementary Figures 15 and 16).

### Sensitivity analysis

First, by excluding low-quality trials of Di Florio et al.[9], outcomes of 3-year (*P* < 0.00001), 5-year (*P* < 0.0001), and 10-year (*P* = 0.05) overall survival and 3-year (*P* < 0.0001), 5-year (*P* = 0.0005), and 10-year (*P* = 0.64) disease-free survival remained stable.

Second, by interchanging statistical modes between a fixed-effects model and a random-effects model, outcomes of 3-year, 5-year, and 10-year overall survival and 3-year, 5-year, and 10-year disease-free survival remained unchanged.

Third, by randomly eliminating included trials on the STATA 14.0 platform, the outcome stability of 3-year and 5-year overall survival and 3-year, 5-year, and 10-year disease-free survival was graphically confirmed. However, the result of 10-year overall survival seems unstable (Supplementary Figure 17).

### Publication bias

The funnel plots of 3-year overall survival and disease-free survival were graphically symmetric. Additionally, the Harbord test (*P* = 0.139 and *P* = 0.192) and the Peters test (*P* = 0.554 and *P* = 0.357) confirmed that no publication bias existed among the included studies (Supplementary Figure 18).

## DISCUSSION

mTOR, a highly conserved serine/threonine protein kinase, promotes cell growth and proliferation through phosphorylation of 4EBP1 and S6k, thus controlling protein translation [41]. Evidence indicates that the disruption of protein synthesis at the level of 4EBP1/eIF4E downstream of mTORC1 stimulates tumor formation. Suppression of 4EBP1 activity and the accompanying activation of cap-dependent translation promotes cell proliferation and correlates with cancer development [42]. p-4EBP1, the inactivated form of 4EBP1, might act as a tumor promoter by converging upstream oncogenic signals and driving the proliferative signal downstream, initiating the synthesis of oncogenic proteins such as cyclin D1, c-myc, or vascular endothelial growth factor (VEGF) [43, 44].

Our quantitative results show that higher expression of p-4EBP1 indicates unfavorable prognosis among cancer patients, irrespective of 3-year, 5-year, and 10-year overall survival and 3-year and 5-year disease-free survival. The present study is the first meta-analysis to systemically study the possible prognostic value of p-4EBP1 overexpression in human malignancies. However, our results showing no obvious difference between 10-year disease-free survival, which is the limit of follow-up duration of many studies, and leading to the insufficient number of cohorts (*n* = 4) and participants (*n* = 621) might be the reasonable interpretation of this puzzling consequence. Furthermore, in a departure from the primary included cohorts, Azim et al. [8], Chen et al. [16], Kasajima et al. [19], Lee et al. [7], and Lee et al. [21] suggested that the prognostic value of p-4EBP1 level among cancer patients remains blurred. A significant difference of TNM stage existed between the two groups in Chen et al. [16] (*P* = 0.001) and Lee et al [7] (*P* < 0.001), and the underexpression group had more advanced TNM stages. Whether these outcomes are simply exceptions or were distorted by the confounding element of different TNM stages awaits further clarification. Moreover, according to Azim et al. [8], the adjuvant treatment received and small number of cases might be reasonable explanations for the different results. Lee et al [21] and Kasajima et al. [19] suggest that all outcomes might result from the small number of participants included in these studies, so the results might be confirmed in large-scale prospective studies.

In-depth perspectives of the negative prognostic significance of p-4EBP1 were supplied by subgroup analyses. In different tumor types, underexpression of p-4EBP1 consistently acted as an indicator of better prognosis among patients with breast cancer, cholangiocarcinoma, esophageal cancer, nasopharyngeal cancer, ovarian cancer, pancreatic cancer, and renal cancer. The prognostic impact of p-4EBP1 on colorectal cancer remains ambiguous. Our pooled results confirmed that lower p-4EBP1 level indicates a better survival prognosis regardless of source region, sex, or mean age. Our outcome shows that the prognostic value of p-4EBP1 overexpression was not distorted despite the variety of TNM stages, except for TNM IV, which means that p-4EBP1 might participate in early phases of tumorigenesis and cancer progression. Total and cytoplasmic p-4EBP1 was verified to correlate with a worse prognosis, whereas nuclear expression was not correlated with an unfavorable outcome prediction. This disparity is probably attributable to the fact that mTOR is predominantly cytoplasmic and phosphorylates 4EBP1 in cytoplasm [45]. When 4EBP1 is phosphorylated, the binding affinity for eIF4E is reduced, which promotes the dissociation of eIF4E from p-4EBP1, attenuating the inhibitory effect on eIF4E-dependent translation initiation, including some oncogenes [46].

Although our meta-analysis was designed and performed rigorously, it has limitations. First, all included cohorts were in retrospective studies, which might cause higher internal heterogeneity. The internal heterogeneity might be explained by the different patient characteristics and the different tumor types or TNM stages, such as in Chen et al. [16] and Lee et al. [7]. Prospective and randomized controlled trials are thus needed for a more persuasive conclusion in future studies. Second, although the total sample size reached 3,980, the included cases were still insufficient to draw a consolidated result, especially for the subgroup analyses and 10-year overall survival, which seems unstable according to sensitivity analysis. In addition, as shown in Figure 1, 17 studies related to p-4EBP1 expression and malignancy prognosis were excluded by our meta-analysis because of the lack of original data. Five of the studies indicated that the p-4EBP1 level had a significant impact on cancer prognosis [47–51], but four studies indicated no impact [52–55]. Another limitation is that bias might be introduced because the survival data were mainly extracted from Kaplan-Meier curves and digitized by GetData Graph Digitizer 2.25 software.

This meta-analysis is the first that confirms the unfavorable influence of p-4EBP1 overexpression in survival expectancy. It correlates with worse prognosis for 3-year and 5-year overall and disease-free survival among cancer patients. Additionally, the prognostic significance is independent of sex, mean age, and resource region. Also, p-4EBP1 Thr37/46 instead of p-4EBP1 Thr70, and total p-4EBP1 but not cytoplasmic or nuclear-positive p-4EBP1, act as prognostic predictors in most cancers, which provide more precise data to guide the clinical evaluations for diagnosis and prognosis. Therefore, we believe that p-4EBP1-directed therapy is a hopeful strategy for cancer patients.

## SUPPLEMENTARY MATERIALS FIGURES AND TABLE




