# Supplementary material for: Meta-analysis of the prognostic value of p-4EBP1 in human malignancies
Source: Oncotarget. 2017 Dec 7;9(2):2761–9. doi: 10.18632/oncotarget.23031 (PMC5788677; doi:10.18632/oncotarget.23031)
Supplement: Supplementary file 1 [file oncotarget-09-2761-s001.pdf]

Meta-analysis of the prognostic value of p-4EBP1 in human malignancies

SUPPLEMENTARY MATERIALS

A

Study or Subgroup	High p-4EBP1 Events	High p-4EBP1 Total	Low p-4EBP1 Events	Low p-4EBP1 Total	Weight	Odds Ratio M-H, Random, 95% CI
Azim 2016	13	19	10	14	5.2%	0.87 [0.19, 3.92]
Benavente 2009	15	25	34	39	6.7%	0.22 [0.06, 0.76]
Campbell 2015	26	43	55	70	10.0%	0.42 [0.18, 0.96]
Fang 2014	2	35	4	26	4.1%	0.33 [0.06, 1.98]
Lee 2015	60	64	95	115	7.5%	3.16 [1.03, 9.69]
Ma 2015	62	74	191	211	10.7%	0.54 [0.25, 1.17]
Malinowsky 2014	18	24	75	78	5.3%	0.12 [0.03, 0.53]
Meric-Bernstam 2012	6	8	165	180	4.4%	0.27 [0.05, 1.47]
Nishikawa 2014	20	40	74	97	10.7%	0.31 [0.14, 0.68]
Nishikawa 2015	4	21	18	28	6.1%	0.13 [0.03, 0.50]
No 2011	38	88	12	15	6.1%	0.19 [0.05, 0.72]
WangS 2014-C1	41	69	11	14	5.9%	0.40 [0.10, 1.56]
WangS 2014-C2	37	58	20	25	7.6%	0.44 [0.14, 1.35]
Zhou 2004	49	70	38	47	9.5%	0.55 [0.23, 1.34]
**Total (95% CI)**		**638**		**959**	**100.0%**	**0.40 [0.27, 0.60]**
Total events	391		802			

Heterogeneity: Tau² = 0.26; Chi² = 23.23, df = 13 (P = 0.04); I² = 44%
Test for overall effect: Z = 4.37 (P < 0.0001)

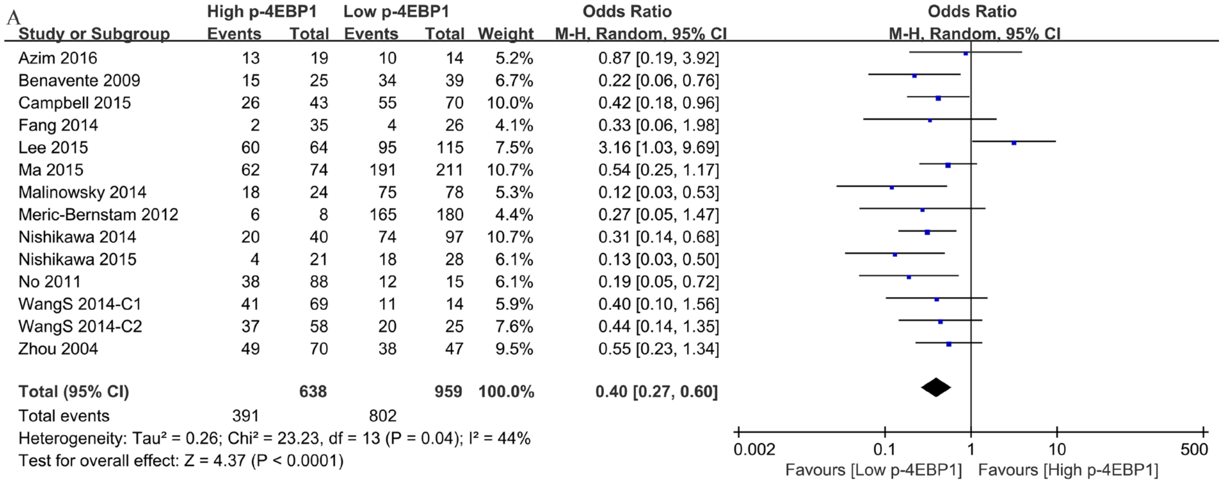

B

Study or Subgroup	High p-4EBP1 Events	High p-4EBP1 Total	Low p-4EBP1 Events	Low p-4EBP1 Total	Weight	Odds Ratio M-H, Random, 95% CI
Azim 2016	13	19	9	14	5.9%	1.20 [0.28, 5.18]
Benavente 2009	13	25	32	39	7.7%	0.24 [0.08, 0.74]
Campbell 2015	23	43	48	70	10.1%	0.53 [0.24, 1.15]
Fang 2014	0	35	3	26	2.1%	0.09 [0.00, 1.92]
Lee 2015	60	64	92	115	7.8%	3.75 [1.24, 11.38]
Ma 2015	57	74	173	211	11.1%	0.74 [0.39, 1.40]
Malinowsky 2014	18	24	73	78	6.8%	0.21 [0.06, 0.75]
Meric-Bernstam 2012	3	8	160	180	5.7%	0.07 [0.02, 0.34]
Nishikawa 2014	17	40	64	97	10.3%	0.38 [0.18, 0.81]
Nishikawa 2015	4	21	16	28	6.6%	0.18 [0.05, 0.66]
WangS 2014-C1	31	69	9	14	7.3%	0.45 [0.14, 1.49]
WangS 2014-C2	29	58	17	25	8.7%	0.47 [0.18, 1.26]
Zhou 2004	40	70	36	47	9.8%	0.41 [0.18, 0.93]
**Total (95% CI)**		**550**		**944**	**100.0%**	**0.44 [0.28, 0.70]**
Total events	308		732			

Heterogeneity: Tau² = 0.40; Chi² = 29.47, df = 12 (P = 0.003); I² = 59%
Test for overall effect: Z = 3.47 (P = 0.0005)

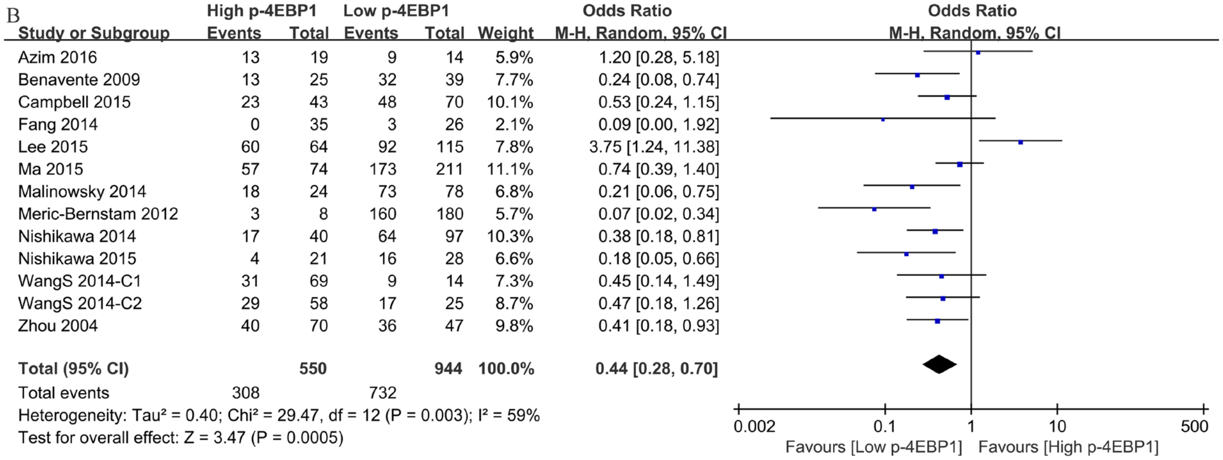

C

Study or Subgroup	High p-4EBP1 Events	High p-4EBP1 Total	Low p-4EBP1 Events	Low p-4EBP1 Total	Weight	Odds Ratio M-H, Random, 95% CI
Lee 2015	60	64	92	115	25.6%	3.75 [1.24, 11.38]
Meric-Bernstam 2012	1	8	128	180	15.9%	0.06 [0.01, 0.48]
Nishikawa 2014	17	40	38	97	29.3%	1.15 [0.54, 2.42]
Zhou 2004	31	70	29	47	29.2%	0.49 [0.23, 1.05]
**Total (95% CI)**		**182**		**439**	**100.0%**	**0.76 [0.23, 2.44]**
Total events	109		287			

Heterogeneity: Tau² = 1.08; Chi² = 15.61, df = 3 (P = 0.001); I² = 81%
Test for overall effect: Z = 0.47 (P = 0.64)

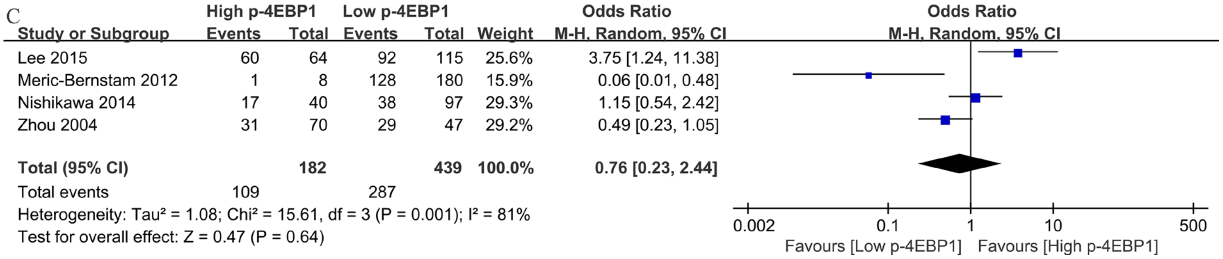

Supplementary Figure 1: The correlation between p-4EBP1 expression and 3-year (**A**), 5-year (**B**), and 10-year (**C**) disease-free survival in malignancies.

Study or Subgroup	High p-4EBP1 Events	High p-4EBP1 Total	Low p-4EBP1 Events	Low p-4EBP1 Total	Weight	Odds Ratio M-H, Random, 95% CI
**1.1.1 Cholangiocarcinoma**						
Fang 2014	4	35	9	26	3.5%	0.24 [0.07, 0.91]
Wang 2012	2	39	7	38	2.8%	0.24 [0.05, 1.24]
**Subtotal (95% CI)**		**74**		**64**	**6.3%**	**0.24 [0.09, 0.68]**
Total events	6		16			
Heterogeneity: Tau² = 0.00; Chi² = 0.00, df = 1 (P = 0.99); I² = 0%						
Test for overall effect: Z = 2.71 (P = 0.007)						
**1.1.2 Colorectal cancer**						
Chen 2017	33	46	39	70	4.9%	2.02 [0.91, 4.47]
Malinowsky 2014	29	32	84	86	2.4%	0.23 [0.04, 1.45]
**Subtotal (95% CI)**		**78**		**156**	**7.4%**	**0.80 [0.10, 6.58]**
Total events	62		123			
Heterogeneity: Tau² = 1.83; Chi² = 4.51, df = 1 (P = 0.03); I² = 78%						
Test for overall effect: Z = 0.20 (P = 0.84)						
**1.1.3 Esophageal cancer**						
Chao 2012	9	26	21	34	4.2%	0.33 [0.11, 0.95]
Yeh 2011	9	38	14	40	4.4%	0.58 [0.21, 1.55]
**Subtotal (95% CI)**		**64**		**74**	**8.5%**	**0.44 [0.21, 0.92]**
Total events	18		35			
Heterogeneity: Tau² = 0.00; Chi² = 0.58, df = 1 (P = 0.45); I² = 0%						
Test for overall effect: Z = 2.20 (P = 0.03)						
**1.1.4 Lung cancer**						
LeeH 2015-C1	16	25	24	48	4.4%	1.78 [0.66, 4.80]
LeeH 2015-C2	8	28	32	45	4.2%	0.16 [0.06, 0.46]
Lv 2015	9	18	45	57	4.0%	0.27 [0.09, 0.82]
Roh 2015	0	77	9	60	1.3%	0.03 [0.00, 0.61]
Trigka 2013	45	71	29	31	3.1%	0.12 [0.03, 0.54]
**Subtotal (95% CI)**		**219**		**241**	**17.0%**	**0.25 [0.07, 0.86]**
Total events	78		139			
Heterogeneity: Tau² = 1.39; Chi² = 17.19, df = 4 (P = 0.002); I² = 77%						
Test for overall effect: Z = 2.20 (P = 0.03)						
**1.1.5 Nasopharyngeal cancer**						
Chen 2010	107	128	84	95	5.0%	0.67 [0.30, 1.46]
Wang 2014	112	181	52	67	5.4%	0.47 [0.24, 0.90]
**Subtotal (95% CI)**		**309**		**162**	**10.4%**	**0.54 [0.33, 0.89]**
Total events	219		136			
Heterogeneity: Tau² = 0.00; Chi² = 0.47, df = 1 (P = 0.49); I² = 0%						
Test for overall effect: Z = 2.41 (P = 0.02)						
**1.1.6 Ovarian cancer**						
Castellvi 2006	24	41	31	34	3.5%	0.14 [0.04, 0.52]
No 2011	66	88	15	15	1.3%	0.10 [0.01, 1.66]
**Subtotal (95% CI)**		**129**		**49**	**4.8%**	**0.13 [0.04, 0.43]**
Total events	90		46			
Heterogeneity: Tau² = 0.00; Chi² = 0.05, df = 1 (P = 0.82); I² = 0%						
Test for overall effect: Z = 3.32 (P = 0.0009)						
**1.1.7 Pancreatic cancer**						
Florio 2011	10	12	16	19	2.3%	0.94 [0.13, 6.63]
Zhou 2017-C1	3	34	34	115	3.7%	0.23 [0.07, 0.81]
Zhou 2017-C2	2	28	21	129	3.1%	0.40 [0.09, 1.79]
**Subtotal (95% CI)**		**74**		**263**	**9.0%**	**0.36 [0.15, 0.86]**
Total events	15		71			
Heterogeneity: Tau² = 0.00; Chi² = 1.44, df = 2 (P = 0.49); I² = 0%						
Test for overall effect: Z = 2.30 (P = 0.02)						
**1.1.8 Others**						
Benavente 2009	17	25	35	39	3.5%	0.24 [0.06, 0.92]
Castellvi 2009	58	76	40	44	3.9%	0.32 [0.10, 1.02]
Cedrés 2012	1	13	2	17	1.6%	0.63 [0.05, 7.75]
El-Habr 2014	26	29	41	41	1.2%	0.09 [0.00, 1.84]
Kasajima 2011-C1	23	24	11	15	1.8%	8.36 [0.83, 83.93]
Kasajima 2011-C2	21	23	13	16	2.3%	2.42 [0.36, 16.50]
Korkolopoulou 2012	14	61	6	13	3.7%	0.35 [0.10, 1.20]
Lee 2015	50	64	80	115	5.2%	1.56 [0.77, 3.19]
Qian 2013	21	25	48	50	2.6%	0.22 [0.04, 1.29]
Qu 2016	6	26	8	10	2.5%	0.07 [0.01, 0.45]
Setsu 2011	45	82	25	30	4.2%	0.24 [0.08, 0.70]
Setsu 2013	39	59	34	40	4.3%	0.34 [0.12, 0.96]
**Subtotal (95% CI)**		**507**		**430**	**36.7%**	**0.45 [0.23, 0.87]**
Total events	321		343			
Heterogeneity: Tau² = 0.76; Chi² = 28.80, df = 11 (P = 0.002); I² = 62%						
Test for overall effect: Z = 2.38 (P = 0.02)						
**Total (95% CI)**		**1454**		**1439**	**100.0%**	**0.40 [0.28, 0.58]**
Total events	809		909			
Heterogeneity: Tau² = 0.53; Chi² = 70.00, df = 29 (P < 0.0001); I² = 59%						
Test for overall effect: Z = 4.92 (P < 0.00001)						
Test for subgroup differences: Chi² = 6.89, df = 7 (P = 0.44), I² = 0%						

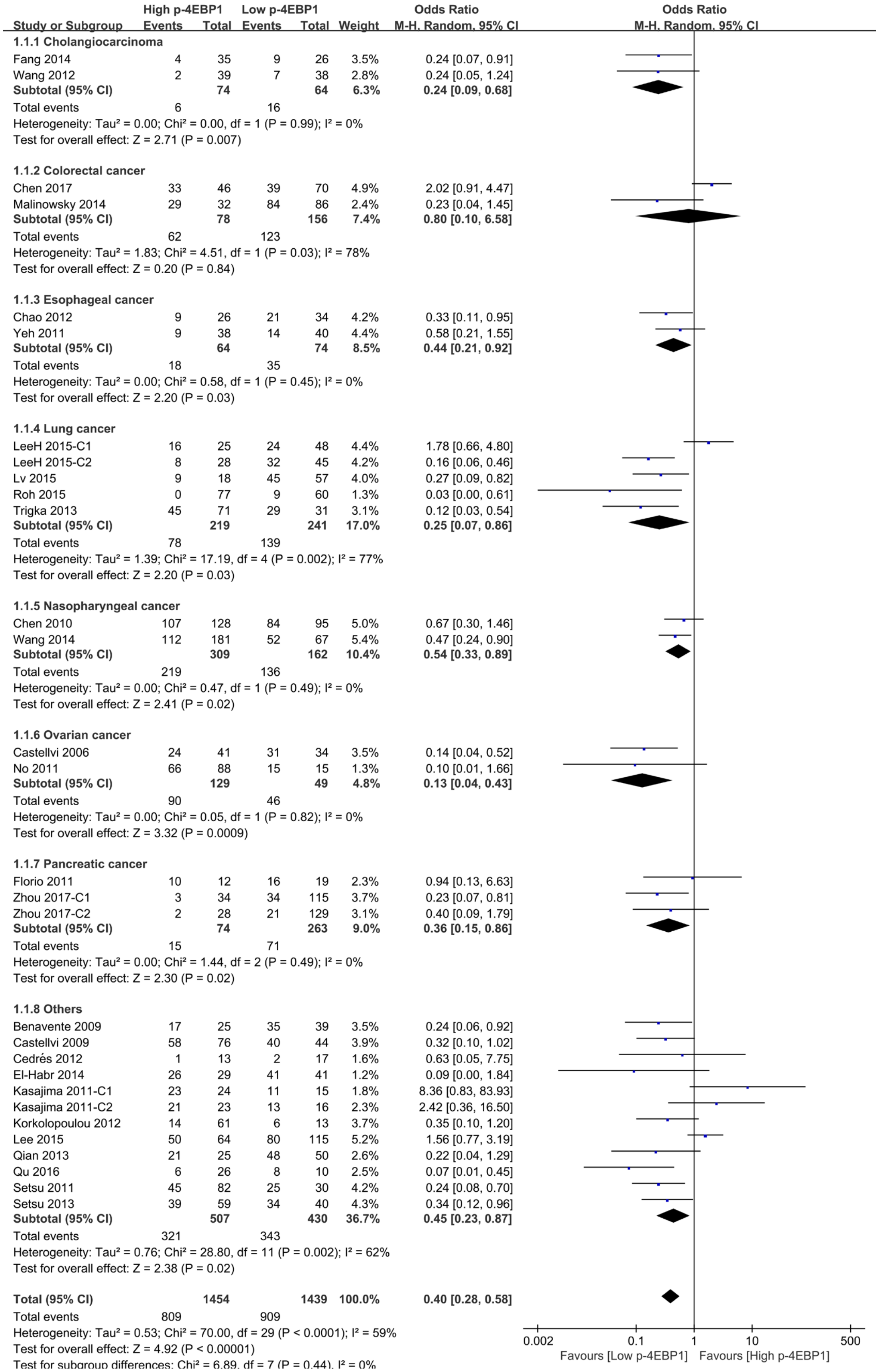

Supplementary Figure 2: The correlation between p-4EBP1 expression and 3-year overall survival based on different cancer types.

Study or Subgroup	High p-4EBP1 Events	High p-4EBP1 Total	Low p-4EBP1 Events	Low p-4EBP1 Total	Weight	Odds Ratio M-H, Random, 95% CI
**1.2.2 Colorectal cancer**						
Chen 2017	25	46	32	70	5.1%	1.41 [0.67, 2.98]
Malinowsky 2014	27	32	80	86	4.2%	0.41 [0.11, 1.43]
**Subtotal (95% CI)**		**78**		**156**	**9.2%**	**0.84 [0.25, 2.82]**
Total events	52		112			
Heterogeneity: Tau² = 0.50; Chi² = 2.79, df = 1 (P = 0.10); I² = 64%						
Test for overall effect: Z = 0.28 (P = 0.78)						
**1.2.3 Esophageal cancer**						
Chao 2012	4	26	20	34	4.2%	0.13 [0.04, 0.45]
Yeh 2011	5	38	13	40	4.4%	0.31 [0.10, 0.99]
**Subtotal (95% CI)**		**64**		**74**	**8.6%**	**0.21 [0.09, 0.50]**
Total events	9		33			
Heterogeneity: Tau² = 0.03; Chi² = 1.08, df = 1 (P = 0.30); I² = 7%						
Test for overall effect: Z = 3.48 (P = 0.0005)						
**1.2.4 Lung cancer**						
LeeH 2015-C1	12	25	17	48	4.7%	1.68 [0.63, 4.50]
LeeH 2015-C2	6	28	24	45	4.5%	0.24 [0.08, 0.70]
Lv 2015	3	18	40	57	4.0%	0.09 [0.02, 0.33]
Trigka 2013	45	71	29	31	3.8%	0.12 [0.03, 0.54]
**Subtotal (95% CI)**		**142**		**181**	**17.0%**	**0.27 [0.07, 1.11]**
Total events	66		110			
Heterogeneity: Tau² = 1.69; Chi² = 16.47, df = 3 (P = 0.0009); I² = 82%						
Test for overall effect: Z = 1.82 (P = 0.07)						
**1.2.5 Nasopharyngeal cancer**						
Chen 2010	77	128	72	95	5.3%	0.48 [0.27, 0.87]
Wang 2014	98	181	48	67	5.3%	0.47 [0.25, 0.86]
**Subtotal (95% CI)**		**309**		**162**	**10.5%**	**0.48 [0.31, 0.72]**
Total events	175		120			
Heterogeneity: Tau² = 0.00; Chi² = 0.01, df = 1 (P = 0.94); I² = 0%						
Test for overall effect: Z = 3.46 (P = 0.0005)						
**1.2.6 Pancreatic cancer**						
Florio 2011	7	12	16	19	3.5%	0.26 [0.05, 1.41]
Zhou 2017-C1	1	34	34	115	3.0%	0.07 [0.01, 0.55]
Zhou 2017-C2	0	28	18	129	2.0%	0.11 [0.01, 1.81]
**Subtotal (95% CI)**		**74**		**263**	**8.5%**	**0.15 [0.04, 0.47]**
Total events	8		68			
Heterogeneity: Tau² = 0.00; Chi² = 1.11, df = 2 (P = 0.58); I² = 0%						
Test for overall effect: Z = 3.21 (P = 0.001)						
**1.2.8 Others**						
Benavente 2009	12	25	29	39	4.5%	0.32 [0.11, 0.92]
Castellvi 2006	6	41	31	34	3.8%	0.02 [0.00, 0.07]
Castellvi 2009	57	76	40	44	4.4%	0.30 [0.09, 0.95]
El-Habr 2014	26	29	41	41	1.9%	0.09 [0.00, 1.84]
Fang 2014	0	35	4	26	1.9%	0.07 [0.00, 1.37]
Kasajima 2011-C1	21	24	9	15	3.6%	4.67 [0.95, 22.90]
Kasajima 2011-C2	21	23	10	16	3.3%	6.30 [1.07, 36.94]
Korkolopoulou 2012	0	61	6	13	1.9%	0.01 [0.00, 0.18]
Lee 2015	50	64	67	115	5.1%	2.56 [1.27, 5.15]
Qian 2013	18	25	44	50	4.3%	0.35 [0.10, 1.19]
Qu 2016	0	26	8	10	1.8%	0.01 [0.00, 0.13]
Setsu 2011	39	82	20	30	4.9%	0.45 [0.19, 1.09]
Setsu 2013	30	59	31	40	4.8%	0.30 [0.12, 0.74]
**Subtotal (95% CI)**		**570**		**473**	**46.2%**	**0.29 [0.11, 0.76]**
Total events	280		340			
Heterogeneity: Tau² = 2.34; Chi² = 78.33, df = 12 (P < 0.00001); I² = 85%						
Test for overall effect: Z = 2.52 (P = 0.01)						
**Total (95% CI)**		**1237**		**1309**	**100.0%**	**0.32 [0.19, 0.53]**
Total events	590		783			
Heterogeneity: Tau² = 1.16; Chi² = 113.96, df = 25 (P < 0.00001); I² = 78%						
Test for overall effect: Z = 4.44 (P < 0.00001)						
Test for subgroup differences: Chi² = 7.61, df = 5 (P = 0.18), I² = 34.3%						

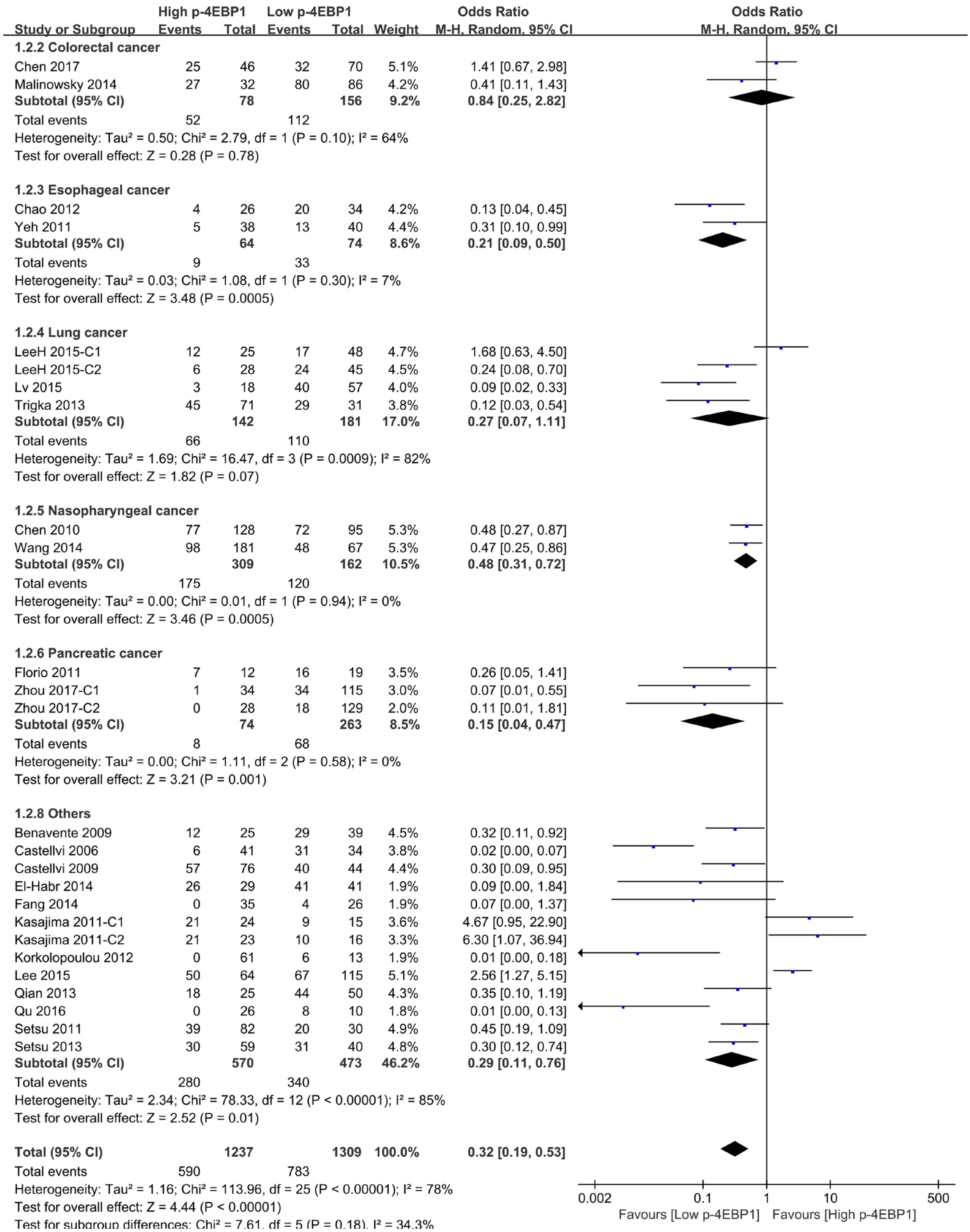

Supplementary Figure 3: The correlation between p-4EBP1 expression and 5-year overall survival based on different cancer types.

A

Study or Subgroup	High p-4EBP1 Events	High p-4EBP1 Total	Low p-4EBP1 Events	Low p-4EBP1 Total	Weight	Odds Ratio M-H, Random, 95% CI
**1.4.1 Breast cancer**						
Azim 2016	13	19	10	14	5.2%	0.87 [0.19, 3.92]
Ma 2015	62	74	191	211	10.7%	0.54 [0.25, 1.17]
Meric-Bernstam 2012	6	8	165	180	4.4%	0.27 [0.05, 1.47]
WangS 2014-C1	41	69	11	14	5.9%	0.40 [0.10, 1.56]
WangS 2014-C2	37	58	20	25	7.6%	0.44 [0.14, 1.35]
Zhou 2004	49	70	38	47	9.5%	0.55 [0.23, 1.34]
**Subtotal (95% CI)**		**298**		**491**	**43.4%**	**0.51 [0.32, 0.79]**
Total events	208		435			
Heterogeneity: Tau² = 0.00; Chi² = 1.25, df = 5 (P = 0.94); I² = 0%						
Test for overall effect: Z = 3.01 (P = 0.003)						
**1.4.2 Renal cancer**						
Campbell 2015	26	43	55	70	10.0%	0.42 [0.18, 0.96]
Nishikawa 2014	20	40	74	97	10.7%	0.31 [0.14, 0.68]
**Subtotal (95% CI)**		**83**		**167**	**20.7%**	**0.36 [0.20, 0.63]**
Total events	46		129			
Heterogeneity: Tau² = 0.00; Chi² = 0.26, df = 1 (P = 0.61); I² = 0%						
Test for overall effect: Z = 3.56 (P = 0.0004)						
**1.4.3 Others**						
Benavente 2009	15	25	34	39	6.7%	0.22 [0.06, 0.76]
Fang 2014	2	35	4	26	4.1%	0.33 [0.06, 1.98]
Lee 2015	60	64	95	115	7.5%	3.16 [1.03, 9.69]
Malinowsky 2014	18	24	75	78	5.3%	0.12 [0.03, 0.53]
Nishikawa 2015	4	21	18	28	6.1%	0.13 [0.03, 0.50]
No 2011	38	88	12	15	6.1%	0.19 [0.05, 0.72]
**Subtotal (95% CI)**		**257**		**301**	**35.9%**	**0.31 [0.10, 0.95]**
Total events	137		238			
Heterogeneity: Tau² = 1.48; Chi² = 20.64, df = 5 (P = 0.0009); I² = 76%						
Test for overall effect: Z = 2.06 (P = 0.04)						
**Total (95% CI)**		**638**		**959**	**100.0%**	**0.40 [0.27, 0.60]**
Total events	391		802			
Heterogeneity: Tau² = 0.26; Chi² = 23.23, df = 13 (P = 0.04); I² = 44%						
Test for overall effect: Z = 4.37 (P < 0.0001)						
Test for subgroup differences: Chi² = 1.28, df = 2 (P = 0.53), I² = 0%						

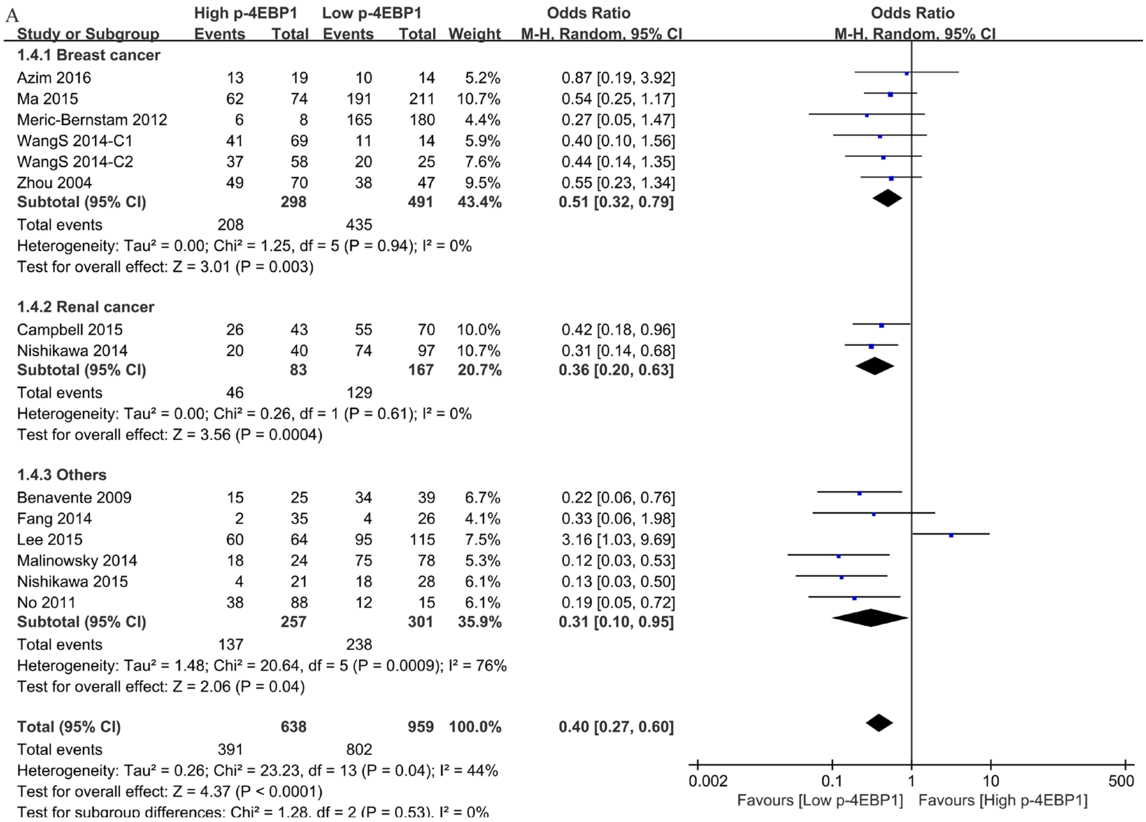

B

Study or Subgroup	High p-4EBP1 Events	High p-4EBP1 Total	Low p-4EBP1 Events	Low p-4EBP1 Total	Weight	Odds Ratio M-H, Random, 95% CI
**1.5.1 Breast cancer**						
Azim 2016	13	19	9	14	6.0%	1.20 [0.28, 5.18]
Ma 2015	57	74	173	211	11.1%	0.74 [0.39, 1.40]
Meric-Bernstam 2012	3	8	160	180	5.8%	0.07 [0.02, 0.34]
WangS 2014-C1	31	69	9	14	7.5%	0.45 [0.14, 1.49]
WangS 2014-C2	29	58	17	25	8.7%	0.47 [0.18, 1.26]
Zhou 2004	40	70	36	47	9.9%	0.41 [0.18, 0.93]
**Subtotal (95% CI)**		**298**		**491**	**49.1%**	**0.47 [0.26, 0.82]**
Total events	173		404			
Heterogeneity: Tau² = 0.22; Chi² = 9.16, df = 5 (P = 0.10); I² = 45%						
Test for overall effect: Z = 2.64 (P = 0.008)						
**1.5.2 Renal cancer**						
Campbell 2015	23	43	48	70	10.1%	0.53 [0.24, 1.15]
Nishikawa 2014	17	40	64	97	10.4%	0.38 [0.18, 0.81]
**Subtotal (95% CI)**		**83**		**167**	**20.5%**	**0.45 [0.26, 0.77]**
Total events	40		112			
Heterogeneity: Tau² = 0.00; Chi² = 0.34, df = 1 (P = 0.56); I² = 0%						
Test for overall effect: Z = 2.92 (P = 0.004)						
**1.5.3 Others**						
Benavente 2009	4	21	16	28	6.7%	0.18 [0.05, 0.66]
Fang 2014	0	35	3	26	2.1%	0.09 [0.00, 1.92]
Lee 2015	60	64	92	115	7.9%	3.75 [1.24, 11.38]
Malinowsky 2014	18	24	73	78	6.9%	0.21 [0.06, 0.75]
Nishikawa 2015	4	21	16	28	6.7%	0.18 [0.05, 0.66]
**Subtotal (95% CI)**		**165**		**275**	**30.4%**	**0.34 [0.08, 1.47]**
Total events	86		200			
Heterogeneity: Tau² = 2.14; Chi² = 20.72, df = 4 (P = 0.0004); I² = 81%						
Test for overall effect: Z = 1.45 (P = 0.15)						
**Total (95% CI)**		**546**		**933**	**100.0%**	**0.43 [0.27, 0.70]**
Total events	299		716			
Heterogeneity: Tau² = 0.42; Chi² = 30.16, df = 12 (P = 0.003); I² = 60%						
Test for overall effect: Z = 3.46 (P = 0.0005)						
Test for subgroup differences: Chi² = 0.16, df = 2 (P = 0.92), I² = 0%						

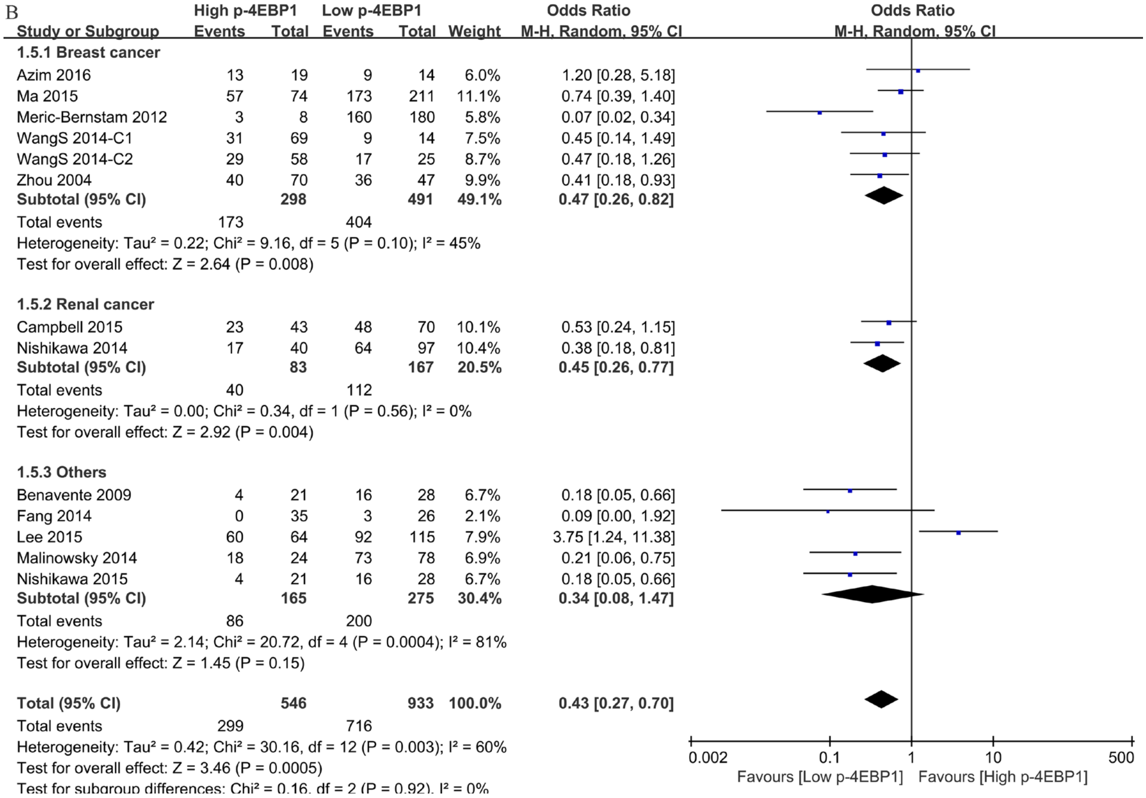

Supplementary Figure 4: The correlation between p-4EBP1 expression and 3-year (**A**) and 5-year (**B**) disease-free survival based on different cancer types.

Study or Subgroup	High p-4EBP1 Events	High p-4EBP1 Total	Low p-4EBP1 Events	Low p-4EBP1 Total	Weight	Odds Ratio M-H, Random, 95% CI
**1.1.1 Asian**						
Chao 2012	9	26	21	34	4.2%	0.33 [0.11, 0.95]
Chen 2010	107	128	84	95	5.0%	0.67 [0.30, 1.46]
Chen 2017	33	46	39	70	4.9%	2.02 [0.91, 4.47]
Fang 2014	4	35	9	26	3.5%	0.24 [0.07, 0.91]
Lee 2015	50	64	80	115	5.2%	1.56 [0.77, 3.19]
LeeH 2015-C1	16	25	24	48	4.4%	1.78 [0.66, 4.80]
LeeH 2015-C2	8	28	32	45	4.2%	0.16 [0.06, 0.46]
Lv 2015	9	18	45	57	4.0%	0.27 [0.09, 0.82]
No 2011	66	88	15	15	1.3%	0.10 [0.01, 1.66]
Qu 2016	6	26	8	10	2.5%	0.07 [0.01, 0.45]
Roh 2015	0	77	9	60	1.3%	0.03 [0.00, 0.61]
Setsu 2011	45	82	25	30	4.2%	0.24 [0.08, 0.70]
Setsu 2013	39	59	34	40	4.3%	0.34 [0.12, 0.96]
Wang 2012	2	39	7	38	2.8%	0.24 [0.05, 1.24]
Wang 2014	112	181	52	67	5.4%	0.47 [0.24, 0.90]
Yeh 2011	9	38	14	40	4.4%	0.58 [0.21, 1.55]
Zhou 2017-C1	3	34	34	115	3.7%	0.23 [0.07, 0.81]
Zhou 2017-C2	2	28	21	129	3.1%	0.40 [0.09, 1.79]
**Subtotal (95% CI)**		**1022**		**1034**	**68.3%**	**0.42 [0.27, 0.65]**
Total events	520		553			
Heterogeneity: Tau² = 0.55; Chi² = 49.44, df = 17 (P < 0.0001); I² = 66%						
Test for overall effect: Z = 3.84 (P = 0.0001)						
**1.1.2 non-Asian**						
Benavente 2009	17	25	35	39	3.5%	0.24 [0.06, 0.92]
Castellvi 2006	24	41	31	34	3.5%	0.14 [0.04, 0.52]
Castellvi 2009	58	76	40	44	3.9%	0.32 [0.10, 1.02]
Cedrés 2012	1	13	2	17	1.6%	0.63 [0.05, 7.75]
El-Habr 2014	26	29	41	41	1.2%	0.09 [0.00, 1.84]
Florio 2011	10	12	16	19	2.3%	0.94 [0.13, 6.63]
Kasajima 2011-C1	23	24	11	15	1.8%	8.36 [0.83, 83.93]
Kasajima 2011-C2	21	23	13	16	2.3%	2.42 [0.36, 16.50]
Korkolopoulou 2012	14	61	6	13	3.7%	0.35 [0.10, 1.20]
Malinowsky 2014	29	32	84	86	2.4%	0.23 [0.04, 1.45]
Qian 2013	21	25	48	50	2.6%	0.22 [0.04, 1.29]
Trigka 2013	45	71	29	31	3.1%	0.12 [0.03, 0.54]
**Subtotal (95% CI)**		**432**		**405**	**31.7%**	**0.36 [0.19, 0.67]**
Total events	289		356			
Heterogeneity: Tau² = 0.43; Chi² = 17.72, df = 11 (P = 0.09); I² = 38%						
Test for overall effect: Z = 3.24 (P = 0.001)						
**Total (95% CI)**		**1454**		**1439**	**100.0%**	**0.40 [0.28, 0.58]**
Total events	809		909			
Heterogeneity: Tau² = 0.53; Chi² = 70.00, df = 29 (P < 0.0001); I² = 59%						
Test for overall effect: Z = 4.92 (P < 0.00001)						
Test for subgroup differences: Chi² = 0.14, df = 1 (P = 0.71), I² = 0%						

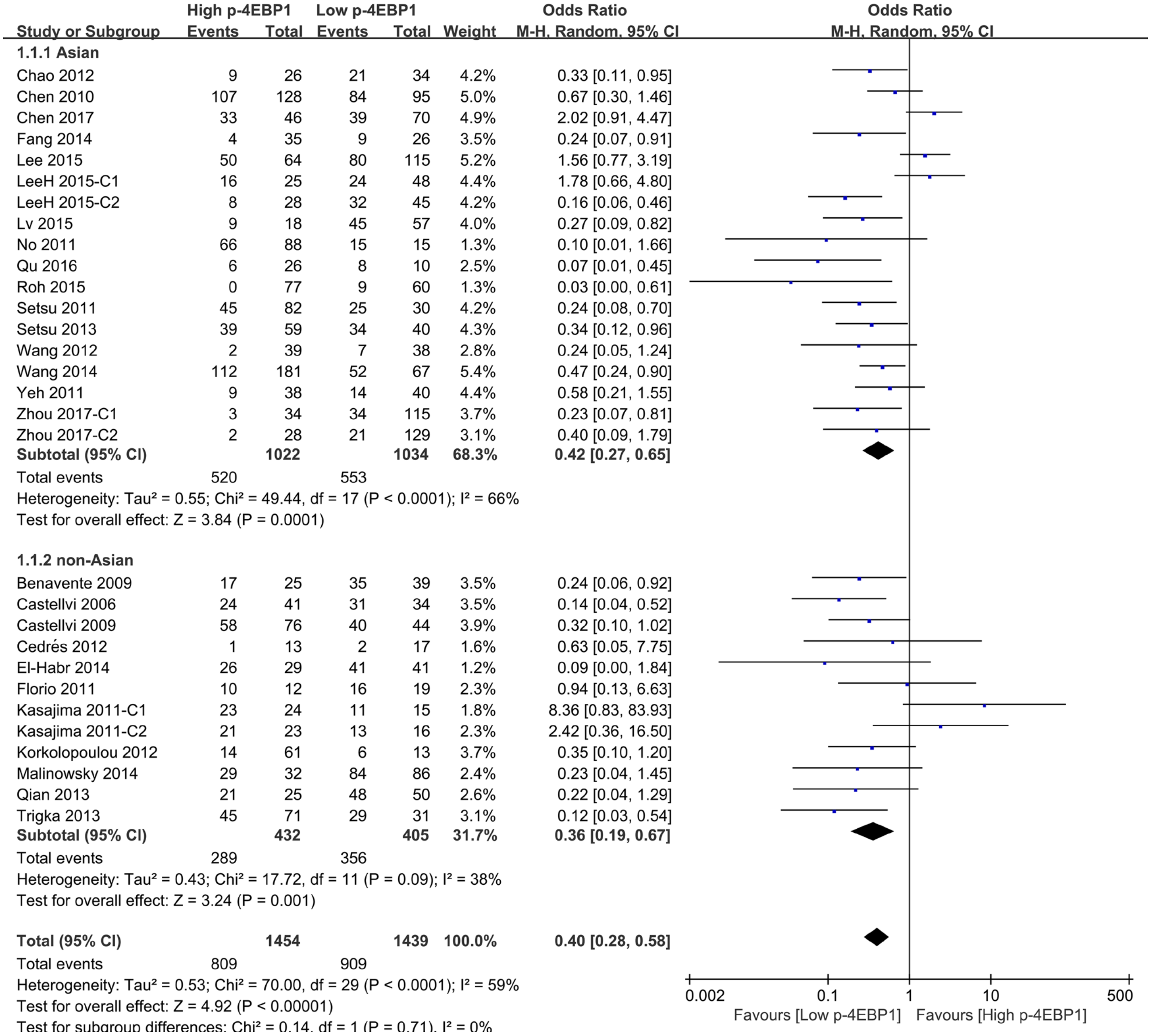

Supplementary Figure 5: The correlation between p-4EBP1 expression and 3-year overall survival based on different regions.

Study or Subgroup	High p-4EBP1 Events	High p-4EBP1 Total	Low p-4EBP1 Events	Low p-4EBP1 Total	Weight	Odds Ratio M-H, Random, 95% CI
**1.2.1 Asian**						
Chao 2012	4	26	20	34	4.2%	0.13 [0.04, 0.45]
Chen 2010	77	128	72	95	5.3%	0.48 [0.27, 0.87]
Chen 2017	25	46	32	70	5.1%	1.41 [0.67, 2.98]
Fang 2014	0	35	4	26	1.9%	0.07 [0.00, 1.37]
Lee 2015	50	64	67	115	5.1%	2.56 [1.27, 5.15]
LeeH 2015-C1	12	25	17	48	4.7%	1.68 [0.63, 4.50]
LeeH 2015-C2	6	28	24	45	4.5%	0.24 [0.08, 0.70]
Lv 2015	3	18	40	57	4.0%	0.09 [0.02, 0.33]
Qu 2016	0	26	8	10	1.8%	0.01 [0.00, 0.13]
Setsu 2011	39	82	20	30	4.9%	0.45 [0.19, 1.09]
Setsu 2013	30	59	31	40	4.8%	0.30 [0.12, 0.74]
Wang 2014	98	181	48	67	5.3%	0.47 [0.25, 0.86]
Yeh 2011	5	38	13	40	4.4%	0.31 [0.10, 0.99]
Zhou 2017-C1	1	34	34	115	3.0%	0.07 [0.01, 0.55]
Zhou 2017-C2	0	28	18	129	2.0%	0.11 [0.01, 1.81]
**Subtotal (95% CI)**		**818**		**921**	**60.8%**	**0.36 [0.20, 0.64]**
Total events	350		448			
Heterogeneity: Tau² = 0.88; Chi² = 63.43, df = 14 (P < 0.00001); I² = 78%						
Test for overall effect: Z = 3.45 (P = 0.0006)						
**1.2.2 non-Asian**						
Benavente 2009	12	25	29	39	4.5%	0.32 [0.11, 0.92]
Castellvi 2006	6	41	31	34	3.8%	0.02 [0.00, 0.07]
Castellvi 2009	57	76	40	44	4.4%	0.30 [0.09, 0.95]
El-Habr 2014	26	29	41	41	1.9%	0.09 [0.00, 1.84]
Florio 2011	7	12	16	19	3.5%	0.26 [0.05, 1.41]
Kasajima 2011-C1	21	24	9	15	3.6%	4.67 [0.95, 22.90]
Kasajima 2011-C2	21	23	10	16	3.3%	6.30 [1.07, 36.94]
Korkolopoulou 2012	0	61	6	13	1.9%	0.01 [0.00, 0.18]
Malinowsky 2014	27	32	80	86	4.2%	0.41 [0.11, 1.43]
Qian 2013	18	25	44	50	4.3%	0.35 [0.10, 1.19]
Trigka 2013	45	71	29	31	3.8%	0.12 [0.03, 0.54]
**Subtotal (95% CI)**		**419**		**388**	**39.2%**	**0.28 [0.10, 0.75]**
Total events	240		335			
Heterogeneity: Tau² = 2.03; Chi² = 45.39, df = 10 (P < 0.00001); I² = 78%						
Test for overall effect: Z = 2.55 (P = 0.01)						
**Total (95% CI)**		**1237**		**1309**	**100.0%**	**0.32 [0.19, 0.53]**
Total events	590		783			
Heterogeneity: Tau² = 1.16; Chi² = 113.96, df = 25 (P < 0.00001); I² = 78%						
Test for overall effect: Z = 4.44 (P < 0.00001)						
Test for subgroup differences: Chi² = 0.19, df = 1 (P = 0.67), I² = 0%						

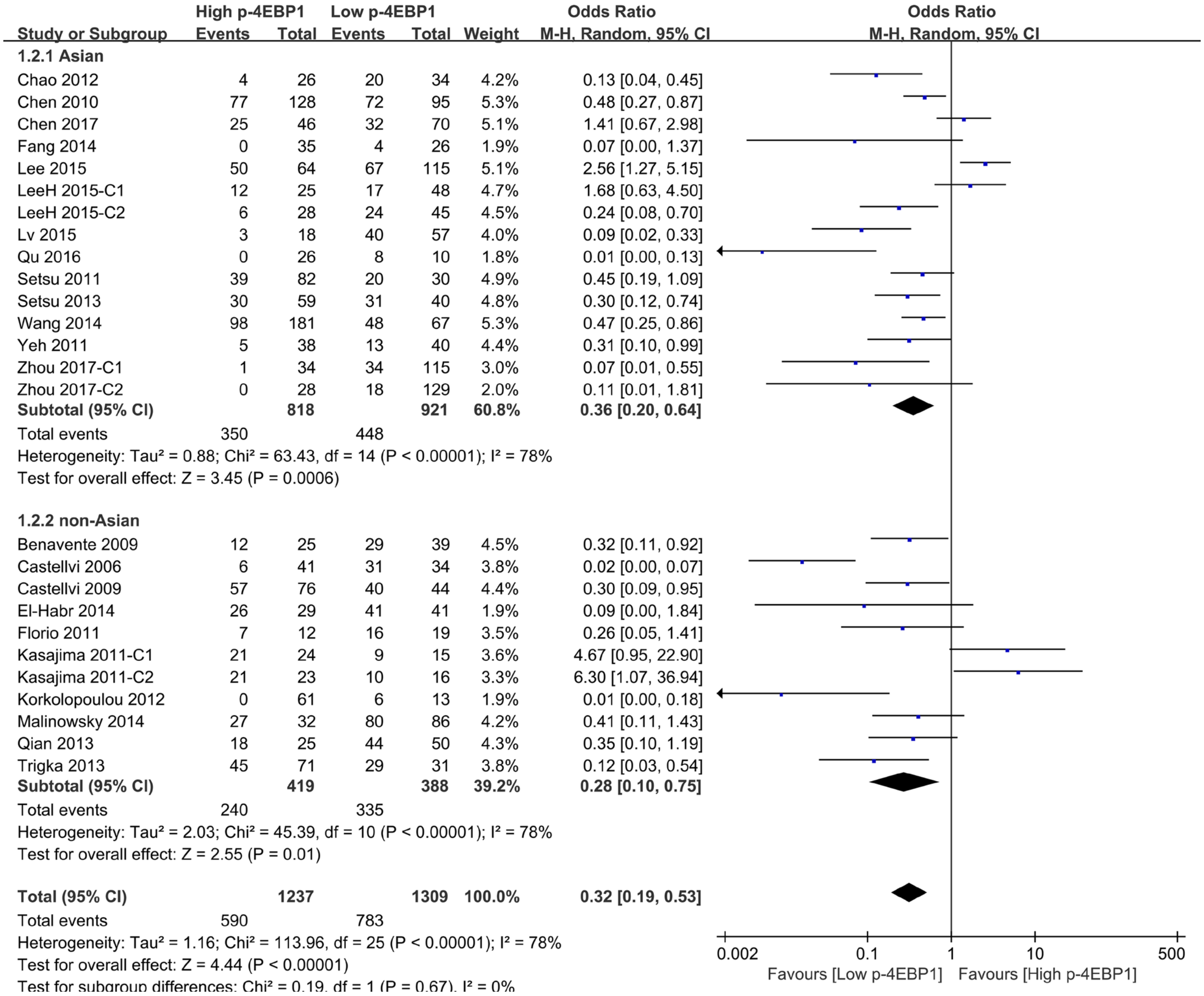

Supplementary Figure 6: The correlation between p-4EBP1 expression and 5-year overall survival based on different regions.

Study or Subgroup	High p-4EBP1 Events	High p-4EBP1 Total	Low p-4EBP1 Events	Low p-4EBP1 Total	Weight	Odds Ratio M-H, Random, 95% CI
**1.1.1 Female-specific**						
Benavente 2009	17	25	35	39	3.4%	0.24 [0.06, 0.92]
Castellvi 2006	24	41	31	34	3.4%	0.14 [0.04, 0.52]
Castellvi 2009	58	76	40	44	3.9%	0.32 [0.10, 1.02]
No 2011	66	88	15	15	1.2%	0.10 [0.01, 1.66]
Qu 2016	6	26	8	10	2.5%	0.07 [0.01, 0.45]
**Subtotal (95% CI)**		**256**		**142**	**14.5%**	**0.19 [0.10, 0.36]**
Total events	171		129			
Heterogeneity: Tau² = 0.00; Chi² = 2.42, df = 4 (P = 0.66); I² = 0%						
Test for overall effect: Z = 4.97 (P < 0.00001)						
**1.1.2 Sex-unspecifc**						
Cedrés 2012	1	13	2	17	1.5%	0.63 [0.05, 7.75]
Chao 2012	9	26	21	34	4.2%	0.33 [0.11, 0.95]
Chen 2010	107	128	84	95	5.1%	0.67 [0.30, 1.46]
Chen 2017	33	46	44	70	5.0%	1.50 [0.67, 3.35]
El-Habr 2014	26	29	41	41	1.1%	0.09 [0.00, 1.84]
Fang 2014	4	35	9	26	3.5%	0.24 [0.07, 0.91]
Florio 2011	10	12	16	19	2.2%	0.94 [0.13, 6.63]
Kasajima 2011-C1	23	24	11	15	1.7%	8.36 [0.83, 83.93]
Kasajima 2011-C2	21	23	13	16	2.3%	2.42 [0.36, 16.50]
Korkolopoulou 2012	14	61	6	13	3.7%	0.35 [0.10, 1.20]
Lee 2015	50	64	80	115	5.3%	1.56 [0.77, 3.19]
LeeH 2015-C1	16	25	24	48	4.4%	1.78 [0.66, 4.80]
LeeH 2015-C2	8	28	32	45	4.3%	0.16 [0.06, 0.46]
Lv 2015	9	18	45	57	4.0%	0.27 [0.09, 0.82]
Malinowsky 2014	29	32	84	86	2.4%	0.23 [0.04, 1.45]
Qian 2013	21	25	48	50	2.5%	0.22 [0.04, 1.29]
Roh 2015	0	77	9	60	1.2%	0.03 [0.00, 0.61]
Setsu 2011	45	82	25	30	4.2%	0.24 [0.08, 0.70]
Setsu 2013	39	59	34	40	4.3%	0.34 [0.12, 0.96]
Trigka 2013	45	71	29	31	3.0%	0.12 [0.03, 0.54]
Wang 2012	2	39	7	38	2.7%	0.24 [0.05, 1.24]
Wang 2014	112	181	52	67	5.5%	0.47 [0.24, 0.90]
Yeh 2011	9	38	14	40	4.4%	0.58 [0.21, 1.55]
Zhou 2017-C1	3	34	34	115	3.7%	0.23 [0.07, 0.81]
Zhou 2017-C2	2	28	21	129	3.0%	0.40 [0.09, 1.79]
**Subtotal (95% CI)**		**1198**		**1297**	**85.5%**	**0.46 [0.32, 0.67]**
Total events	638		785			
Heterogeneity: Tau² = 0.45; Chi² = 54.53, df = 24 (P = 0.0004); I² = 56%						
Test for overall effect: Z = 4.07 (P < 0.0001)						
**Total (95% CI)**		**1454**		**1439**	**100.0%**	**0.40 [0.28, 0.57]**
Total events	809		914			
Heterogeneity: Tau² = 0.47; Chi² = 65.17, df = 29 (P = 0.0001); I² = 55%						
Test for overall effect: Z = 5.14 (P < 0.00001)						
Test for subgroup differences: Chi² = 5.30, df = 1 (P = 0.02), I² = 81.1%						

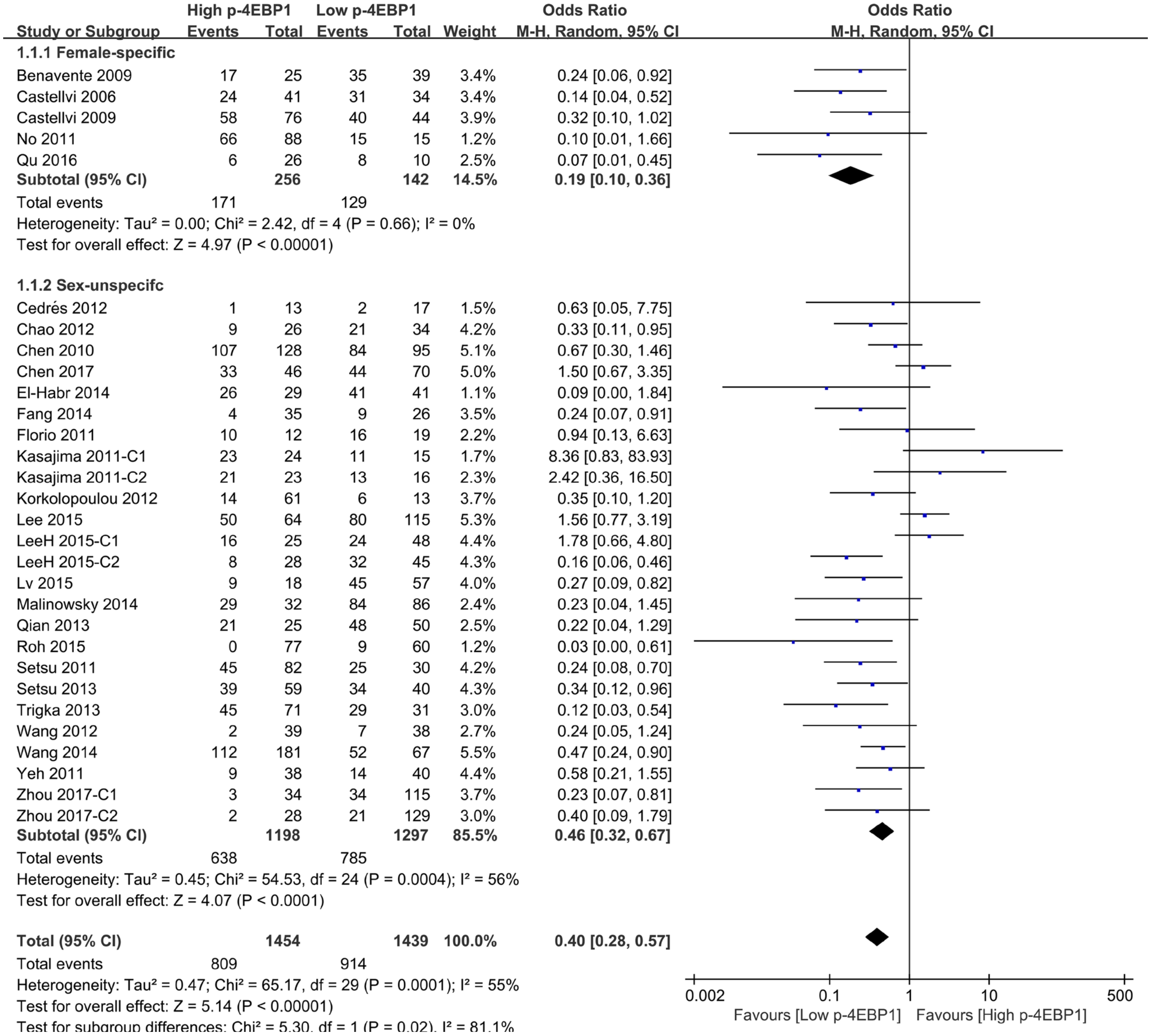

Supplementary Figure 7: The correlation between p-4EBP1 expression and 3-year overall survival based on different sex ratio.

Study or Subgroup	High p-4EBP1 Events	High p-4EBP1 Total	Low p-4EBP1 Events	Low p-4EBP1 Total	Weight	Odds Ratio M-H, Random, 95% CI
**1.2.1 Female-specific**						
Benavente 2009	12	25	29	39	4.5%	0.32 [0.11, 0.92]
Castellvi 2006	6	41	31	34	3.8%	0.02 [0.00, 0.07]
Castellvi 2009	57	76	40	44	4.4%	0.30 [0.09, 0.95]
Qu 2016	0	26	8	10	1.8%	0.01 [0.00, 0.13]
**Subtotal (95% CI)**		**168**		**127**	**14.5%**	**0.08 [0.01, 0.42]**
Total events	75		108			
Heterogeneity: Tau² = 2.32; Chi² = 16.27, df = 3 (P = 0.0010); I² = 82%						
Test for overall effect: Z = 2.95 (P = 0.003)						
**1.2.2 Sex-unspecific**						
Chao 2012	4	26	20	34	4.2%	0.13 [0.04, 0.45]
Chen 2010	77	128	72	95	5.3%	0.48 [0.27, 0.87]
Chen 2017	25	46	32	70	5.1%	1.41 [0.67, 2.98]
El-Habr 2014	26	29	41	41	1.9%	0.09 [0.00, 1.84]
Fang 2014	0	35	4	26	1.9%	0.07 [0.00, 1.37]
Florio 2011	7	12	16	19	3.5%	0.26 [0.05, 1.41]
Kasajima 2011-C1	21	24	9	15	3.6%	4.67 [0.95, 22.90]
Kasajima 2011-C2	21	23	10	16	3.3%	6.30 [1.07, 36.94]
Korkolopoulou 2012	0	61	6	13	1.9%	0.01 [0.00, 0.18]
Lee 2015	50	64	67	115	5.1%	2.56 [1.27, 5.15]
LeeH 2015-C1	12	25	17	48	4.7%	1.68 [0.63, 4.50]
LeeH 2015-C2	6	28	24	45	4.5%	0.24 [0.08, 0.70]
Lv 2015	3	18	40	57	4.0%	0.09 [0.02, 0.33]
Malinowsky 2014	27	32	80	86	4.2%	0.41 [0.11, 1.43]
Qian 2013	18	25	44	50	4.3%	0.35 [0.10, 1.19]
Setsu 2011	39	82	20	30	4.9%	0.45 [0.19, 1.09]
Setsu 2013	30	59	31	40	4.8%	0.30 [0.12, 0.74]
Trigka 2013	45	71	29	31	3.8%	0.12 [0.03, 0.54]
Wang 2014	98	181	48	67	5.3%	0.47 [0.25, 0.86]
Yeh 2011	5	38	13	40	4.4%	0.31 [0.10, 0.99]
Zhou 2017-C1	1	34	34	115	3.0%	0.07 [0.01, 0.55]
Zhou 2017-C2	0	28	18	129	2.0%	0.11 [0.01, 1.81]
**Subtotal (95% CI)**		**1069**		**1182**	**85.5%**	**0.41 [0.25, 0.68]**
Total events	515		675			
Heterogeneity: Tau² = 0.92; Chi² = 83.30, df = 21 (P < 0.00001); I² = 75%						
Test for overall effect: Z = 3.50 (P = 0.0005)						
**Total (95% CI)**		**1237**		**1309**	**100.0%**	**0.32 [0.19, 0.53]**
Total events	590		783			
Heterogeneity: Tau² = 1.16; Chi² = 113.96, df = 25 (P < 0.00001); I² = 78%						
Test for overall effect: Z = 4.44 (P < 0.00001)						
Test for subgroup differences: Chi² = 3.43, df = 1 (P = 0.06), I² = 70.9%						

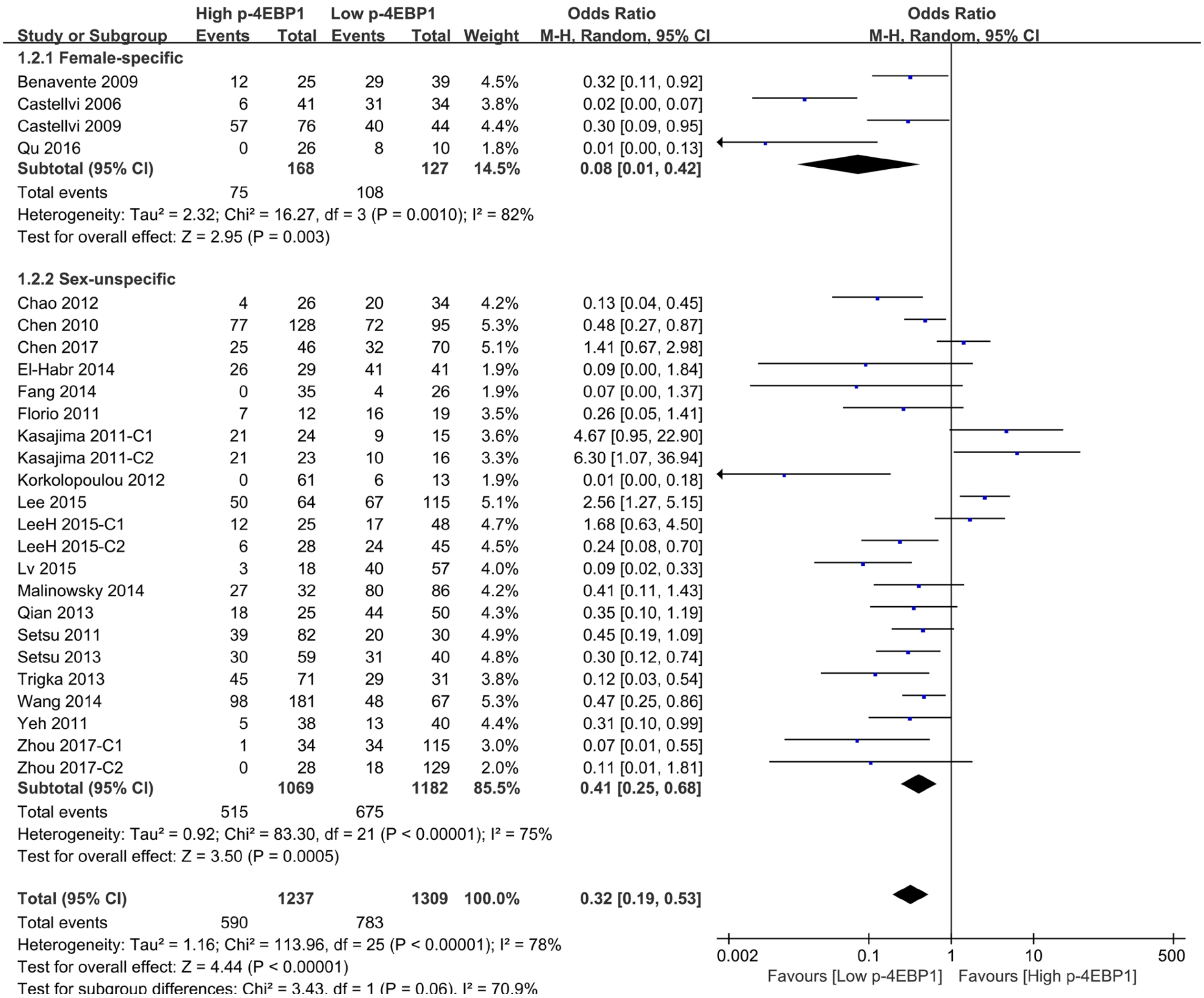

Supplementary Figure 8: The correlation between p-4EBP1 expression and 5-year overall survival based on different sex ratio.

Study or Subgroup	High p-4EBP1 Events	High p-4EBP1 Total	Low p-4EBP1 Events	Low p-4EBP1 Total	Weight	Odds Ratio M-H, Random, 95% CI
**1.1.1 Mean-age<60**						
Benavente 2009	17	25	35	39	4.1%	0.24 [0.06, 0.92]
Castellvi 2006	24	41	31	34	4.1%	0.14 [0.04, 0.52]
Chao 2012	9	26	21	34	5.0%	0.33 [0.11, 0.95]
Chen 2010	107	128	84	95	6.1%	0.67 [0.30, 1.46]
Fang 2014	4	35	9	26	4.2%	0.24 [0.07, 0.91]
Kasajima 2011-Cytoplasm	23	24	11	15	2.1%	8.36 [0.83, 83.93]
Kasajima 2011-Nucleus	21	23	13	16	2.7%	2.42 [0.36, 16.50]
Korkolopoulou 2012	14	61	6	13	4.4%	0.35 [0.10, 1.20]
Lv 2015	9	18	45	57	4.8%	0.27 [0.09, 0.82]
No 2011	66	88	15	15	1.5%	0.10 [0.01, 1.66]
Qian 2013	21	25	48	50	3.0%	0.22 [0.04, 1.29]
Qu 2016	6	26	8	10	2.9%	0.07 [0.01, 0.45]
Wang 2014	112	181	52	67	6.6%	0.47 [0.24, 0.90]
Yeh 2011	9	38	14	40	5.3%	0.58 [0.21, 1.55]
**Subtotal (95% CI)**		**739**		**511**	**56.7%**	**0.38 [0.25, 0.58]**
Total events	442		392			
Heterogeneity: Tau² = 0.24; Chi² = 21.44, df = 13 (P = 0.06); I² = 39%						
Test for overall effect: Z = 4.40 (P < 0.0001)						
**1.1.2 Mean-age>60**						
Castellvi 2009	58	76	40	44	4.7%	0.32 [0.10, 1.02]
Cedrés 2012	1	13	2	17	1.8%	0.63 [0.05, 7.75]
El-Habr 2014	26	29	41	41	1.4%	0.09 [0.00, 1.84]
Lee 2015	50	64	80	115	6.4%	1.56 [0.77, 3.19]
LeeH 2015- Thr37/46	16	25	24	48	5.3%	1.78 [0.66, 4.80]
LeeH 2015- Thr70	8	28	32	45	5.1%	0.16 [0.06, 0.46]
Malinowsky 2014	29	32	84	86	2.8%	0.23 [0.04, 1.45]
Roh 2015	29	32	84	86	2.8%	0.23 [0.04, 1.45]
Setsu 2011	0	77	9	60	1.5%	0.03 [0.00, 0.61]
Trigka 2013	45	71	29	31	3.6%	0.12 [0.03, 0.54]
Zhou 2017-C1	3	34	34	115	4.4%	0.23 [0.07, 0.81]
Zhou 2017-C2	2	28	21	129	3.6%	0.40 [0.09, 1.79]
**Subtotal (95% CI)**		**509**		**817**	**43.3%**	**0.34 [0.17, 0.69]**
Total events	267		480			
Heterogeneity: Tau² = 0.91; Chi² = 32.41, df = 11 (P = 0.0007); I² = 66%						
Test for overall effect: Z = 3.00 (P = 0.003)						
**Total (95% CI)**		**1248**		**1328**	**100.0%**	**0.37 [0.25, 0.54]**
Total events	709		872			
Heterogeneity: Tau² = 0.47; Chi² = 54.39, df = 25 (P = 0.0006); I² = 54%						
Test for overall effect: Z = 5.06 (P < 0.00001)						
Test for subgroup differences: Chi² = 0.07, df = 1 (P = 0.80), I² = 0%						

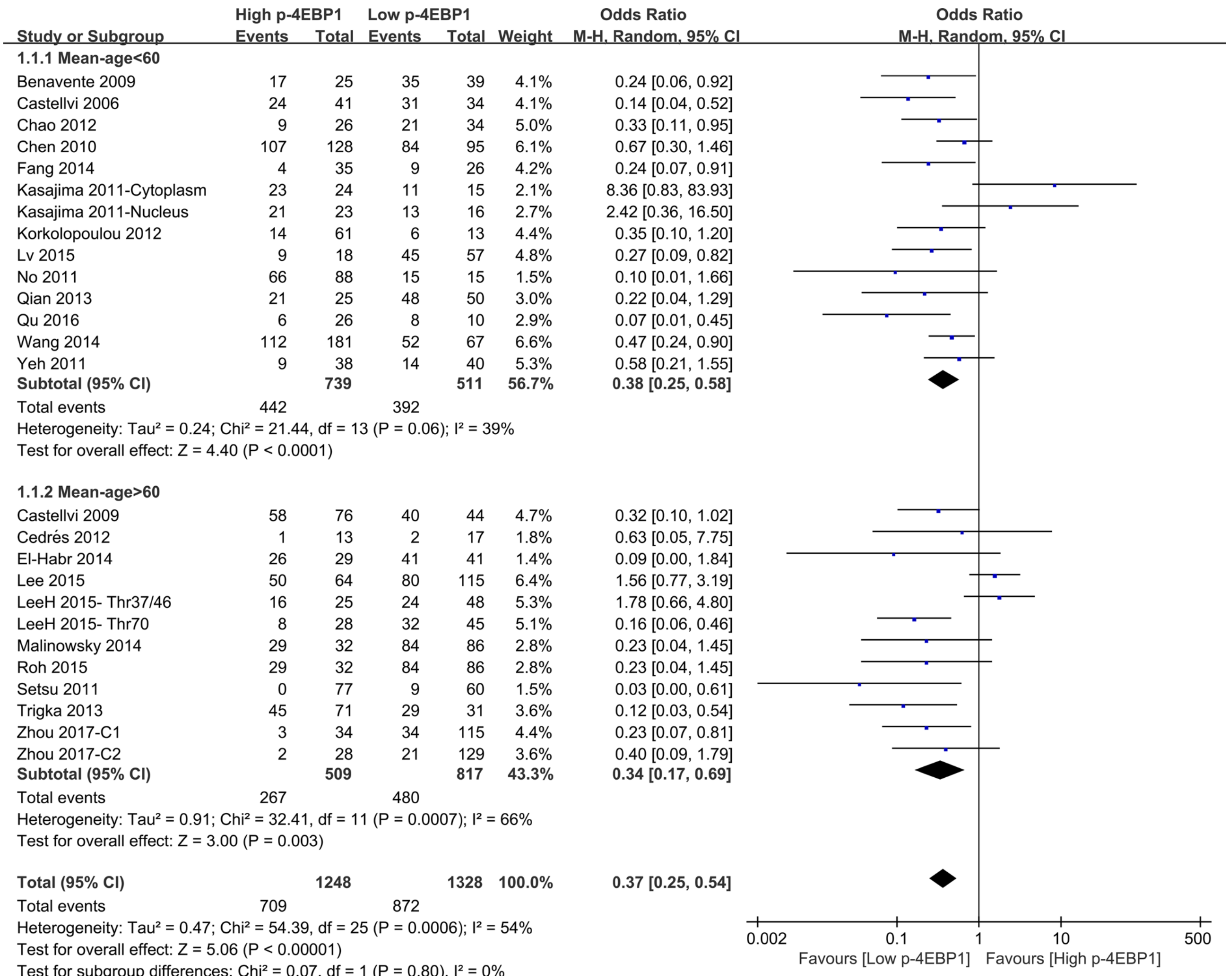

Supplementary Figure 9: The correlation between p-4EBP1 expression and 3-year overall survival based on different mean age.

Study or Subgroup	High p-4EBP1 Events	High p-4EBP1 Total	Low p-4EBP1 Events	Low p-4EBP1 Total	Weight	Odds Ratio M-H, Random, 95% CI
**1.2.1 Mean-age<60**						
Benavente 2009	12	25	29	39	5.2%	0.32 [0.11, 0.92]
Castellvi 2006	6	41	31	34	4.4%	0.02 [0.00, 0.07]
Chao 2012	4	26	20	34	4.8%	0.13 [0.04, 0.45]
Chen 2010	77	128	72	95	6.0%	0.48 [0.27, 0.87]
Fang 2014	0	35	4	26	2.3%	0.07 [0.00, 1.37]
Kasajima 2011-Cytoplasm	21	24	9	15	4.2%	4.67 [0.95, 22.90]
Kasajima 2011-Nucleus	21	23	10	16	3.9%	6.30 [1.07, 36.94]
Korkolopoulou 2012	0	61	6	13	2.3%	0.01 [0.00, 0.18]
Lv 2015	3	18	40	57	4.6%	0.09 [0.02, 0.33]
Qian 2013	18	25	44	50	4.9%	0.35 [0.10, 1.19]
Qu 2016	0	26	8	10	2.1%	0.01 [0.00, 0.13]
Wang 2014	98	181	48	67	5.9%	0.47 [0.25, 0.86]
Yeh 2011	5	38	13	40	5.0%	0.31 [0.10, 0.99]
**Subtotal (95% CI)**		**651**		**496**	**55.8%**	**0.24 [0.11, 0.52]**
Total events	265		334			
Heterogeneity: Tau² = 1.40; Chi² = 59.45, df = 12 (P < 0.00001); I² = 80%						
Test for overall effect: Z = 3.63 (P = 0.0003)						
**1.2.2 Mean-age>60**						
Castellvi 2009	57	76	40	44	5.0%	0.30 [0.09, 0.95]
El-Habr 2014	26	29	41	41	2.3%	0.09 [0.00, 1.84]
Lee 2015	50	64	67	115	5.8%	2.56 [1.27, 5.15]
LeeH 2015- Thr37/46	12	25	17	48	5.3%	1.68 [0.63, 4.50]
LeeH 2015- Thr70	6	28	24	45	5.2%	0.24 [0.08, 0.70]
Malinowsky 2014	27	32	80	86	4.8%	0.41 [0.11, 1.43]
Setsu 2011	39	82	20	30	5.5%	0.45 [0.19, 1.09]
Trigka 2013	45	71	29	31	4.4%	0.12 [0.03, 0.54]
Zhou 2017-C1	1	34	34	115	3.5%	0.07 [0.01, 0.55]
Zhou 2017-C2	0	28	18	129	2.4%	0.11 [0.01, 1.81]
**Subtotal (95% CI)**		**469**		**684**	**44.2%**	**0.38 [0.17, 0.86]**
Total events	263		370			
Heterogeneity: Tau² = 1.18; Chi² = 37.88, df = 9 (P < 0.0001); I² = 76%						
Test for overall effect: Z = 2.31 (P = 0.02)						
**Total (95% CI)**		**1120**		**1180**	**100.0%**	**0.29 [0.17, 0.51]**
Total events	528		704			
Heterogeneity: Tau² = 1.28; Chi² = 103.83, df = 22 (P < 0.00001); I² = 79%						
Test for overall effect: Z = 4.29 (P < 0.0001)						
Test for subgroup differences: Chi² = 0.67, df = 1 (P = 0.41), I² = 0%						

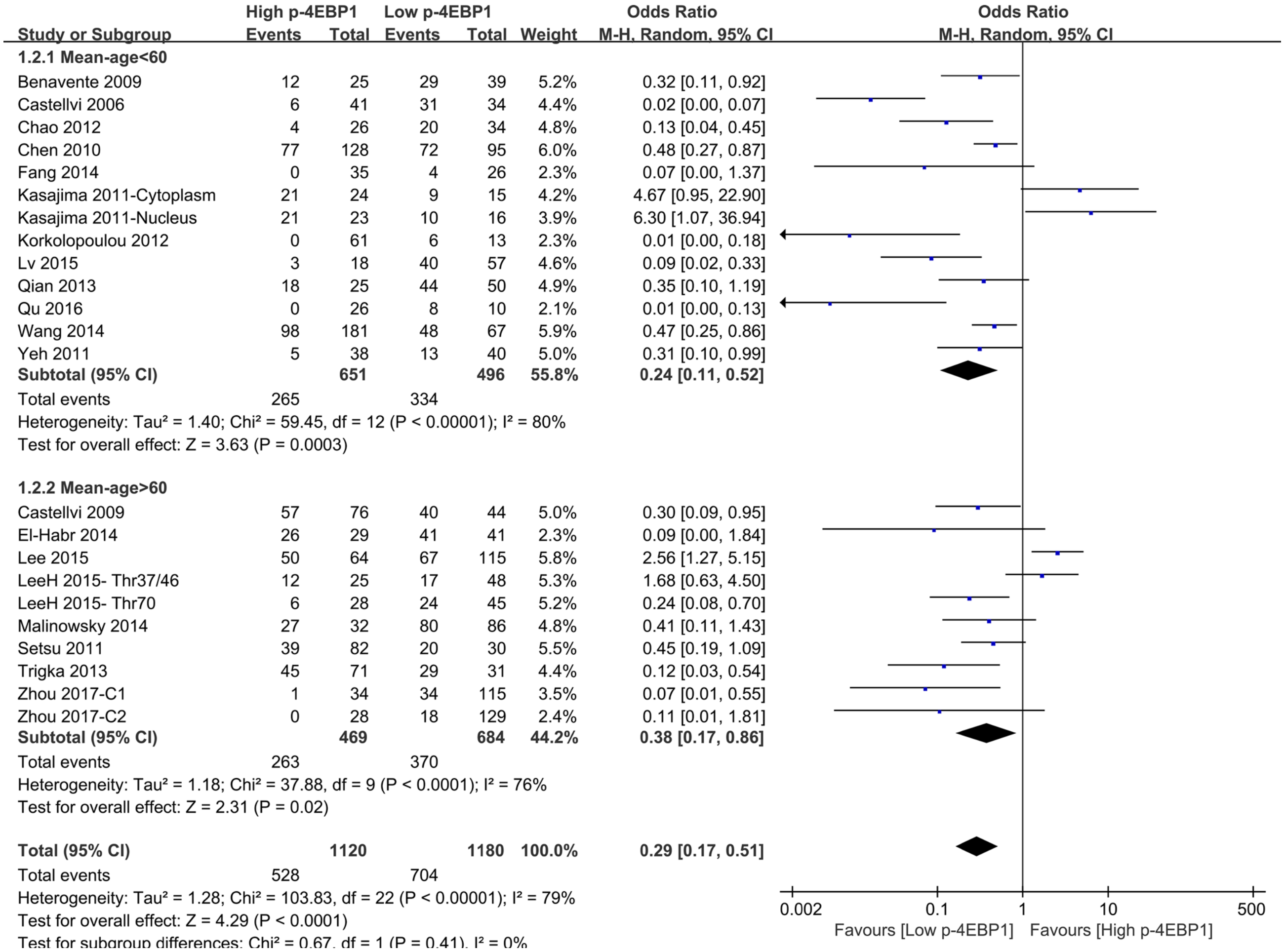

Supplementary Figure 10: The correlation between p-4EBP1 expression and 5-year overall survival based on different mean age.

Study or Subgroup	High p-4EBP1 Events	High p-4EBP1 Total	Low p-4EBP1 Events	Low p-4EBP1 Total	Weight	Odds Ratio M-H, Random, 95% CI
**1.1.1 TNM Ⅰ - Ⅳ**						
Castellvi 2006	24	41	31	34	3.7%	0.14 [0.04, 0.52]
Castellvi 2009	58	76	40	44	4.2%	0.32 [0.10, 1.02]
Chen 2010	107	128	84	95	5.2%	0.67 [0.30, 1.46]
Chen 2017	33	46	39	70	5.2%	2.02 [0.91, 4.47]
El-Habr 2014	26	29	41	41	1.3%	0.09 [0.00, 1.84]
Fang 2014	4	35	9	26	3.8%	0.24 [0.07, 0.91]
Lee 2015	50	64	80	115	5.4%	1.56 [0.77, 3.19]
LeeH 2015-C1	16	25	24	48	4.6%	1.78 [0.66, 4.80]
LeeH 2015-C2	8	28	32	45	4.5%	0.16 [0.06, 0.46]
Lv 2015	9	18	45	57	4.3%	0.27 [0.09, 0.82]
No 2011	66	88	15	15	1.4%	0.10 [0.01, 1.66]
Qu 2016	6	26	8	10	2.7%	0.07 [0.01, 0.45]
Roh 2015	0	77	9	60	1.4%	0.03 [0.00, 0.61]
Setsu 2011	45	82	25	30	4.5%	0.24 [0.08, 0.70]
Wang 2014	112	181	52	67	5.6%	0.47 [0.24, 0.90]
Yeh 2011	9	38	14	40	4.6%	0.58 [0.21, 1.55]
**Subtotal (95% CI)**		**982**		**797**	**62.5%**	**0.40 [0.24, 0.67]**
Total events	573		548			
Heterogeneity: Tau² = 0.71; Chi² = 51.25, df = 15 (P < 0.00001); I² = 71%						
Test for overall effect: Z = 3.47 (P = 0.0005)						
**1.1.2 TNM Ⅰ - Ⅲ**						
Benavente 2009	17	25	35	39	3.7%	0.24 [0.06, 0.92]
Trigka 2013	45	71	29	31	3.3%	0.12 [0.03, 0.54]
Wang 2012	2	39	7	38	3.0%	0.24 [0.05, 1.24]
**Subtotal (95% CI)**		**135**		**108**	**10.0%**	**0.19 [0.08, 0.45]**
Total events	64		71			
Heterogeneity: Tau² = 0.00; Chi² = 0.59, df = 2 (P = 0.75); I² = 0%						
Test for overall effect: Z = 3.78 (P = 0.0002)						
**1.1.3 TNM Ⅰ - Ⅱ**						
Zhou 2017-C1	3	34	34	115	3.9%	0.23 [0.07, 0.81]
Zhou 2017-C2	2	28	21	129	3.3%	0.40 [0.09, 1.79]
**Subtotal (95% CI)**		**62**		**244**	**7.2%**	**0.29 [0.11, 0.75]**
Total events	5		55			
Heterogeneity: Tau² = 0.00; Chi² = 0.29, df = 1 (P = 0.59); I² = 0%						
Test for overall effect: Z = 2.54 (P = 0.01)						
**1.1.4 TNM Ⅱ - Ⅳ**						
Chao 2012	9	26	21	34	4.4%	0.33 [0.11, 0.95]
Korkolopoulou 2012	14	61	6	13	3.9%	0.35 [0.10, 1.20]
Setsu 2013	39	59	34	40	4.6%	0.34 [0.12, 0.96]
**Subtotal (95% CI)**		**146**		**87**	**12.9%**	**0.34 [0.18, 0.64]**
Total events	62		61			
Heterogeneity: Tau² = 0.00; Chi² = 0.01, df = 2 (P = 1.00); I² = 0%						
Test for overall effect: Z = 3.34 (P = 0.0008)						
**1.1.6 TNM Ⅳ**						
Kasajima 2011-C1	23	24	11	15	2.0%	8.36 [0.83, 83.93]
Kasajima 2011-C2	21	23	13	16	2.5%	2.42 [0.36, 16.50]
Qian 2013	21	25	48	50	2.8%	0.22 [0.04, 1.29]
**Subtotal (95% CI)**		**72**		**81**	**7.3%**	**1.50 [0.18, 12.43]**
Total events	65		72			
Heterogeneity: Tau² = 2.46; Chi² = 6.79, df = 2 (P = 0.03); I² = 71%						
Test for overall effect: Z = 0.38 (P = 0.71)						
**Total (95% CI)**		**1397**		**1317**	**100.0%**	**0.39 [0.27, 0.58]**
Total events	769		807			
Heterogeneity: Tau² = 0.58; Chi² = 68.90, df = 26 (P < 0.00001); I² = 62%						
Test for overall effect: Z = 4.74 (P < 0.00001)						
Test for subgroup differences: Chi² = 4.05, df = 4 (P = 0.40), I² = 1.2%						

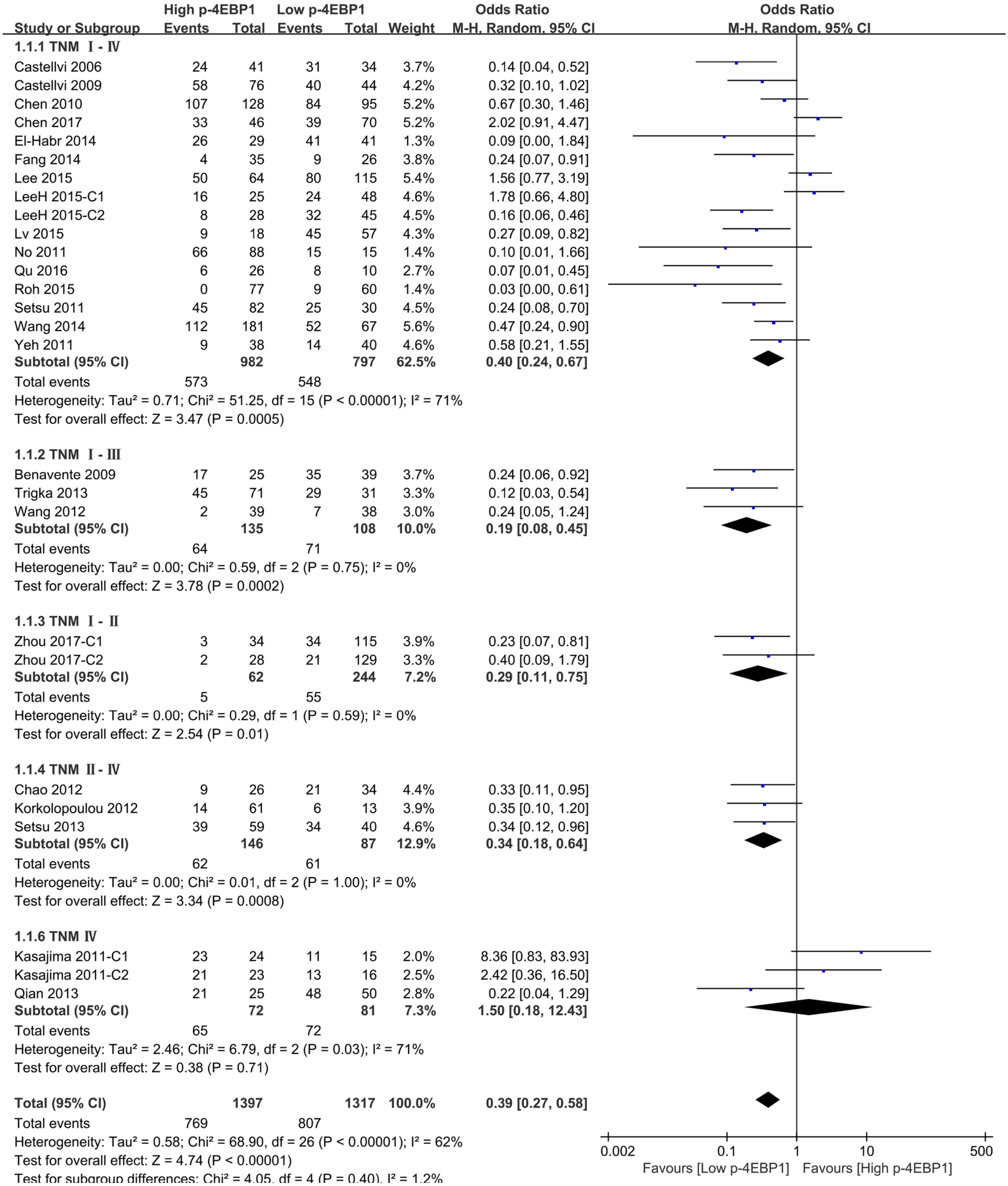

Supplementary Figure 11: The correlation between p-4EBP1 expression and 3-year overall survival based on different TNM stages.

Study or Subgroup	High p-4EBP1 Events	High p-4EBP1 Total	Low p-4EBP1 Events	Low p-4EBP1 Total	Weight	Odds Ratio M-H, Random, 95% CI
**1.2.1 TNM Ⅰ - Ⅳ**						
Castellvi 2006	6	41	31	34	4.2%	0.02 [0.00, 0.07]
Castellvi 2009	57	76	40	44	4.7%	0.30 [0.09, 0.95]
Chen 2010	77	128	72	95	5.6%	0.48 [0.27, 0.87]
Chen 2017	25	46	32	70	5.4%	1.41 [0.67, 2.98]
El-Habr 2014	26	29	41	41	2.1%	0.09 [0.00, 1.84]
Fang 2014	0	35	4	26	2.1%	0.07 [0.00, 1.37]
Lee 2015	50	64	67	115	5.5%	2.56 [1.27, 5.15]
LeeH 2015-C1	12	25	17	48	5.0%	1.68 [0.63, 4.50]
LeeH 2015-C2	6	28	24	45	4.9%	0.24 [0.08, 0.70]
Lv 2015	3	18	40	57	4.4%	0.09 [0.02, 0.33]
Qu 2016	0	26	8	10	2.0%	0.01 [0.00, 0.13]
Setsu 2011	39	82	20	30	5.2%	0.45 [0.19, 1.09]
Wang 2014	98	181	48	67	5.6%	0.47 [0.25, 0.86]
Yeh 2011	5	38	13	40	4.7%	0.31 [0.10, 0.99]
**Subtotal (95% CI)**		**817**		**722**	**61.5%**	**0.32 [0.16, 0.64]**
Total events	404		457			
Heterogeneity: Tau² = 1.19; Chi² = 75.74, df = 13 (P < 0.00001); I² = 83%						
Test for overall effect: Z = 3.27 (P = 0.001)						
**1.2.2 TNM Ⅰ - Ⅲ**						
Benavente 2009	12	25	29	39	4.9%	0.32 [0.11, 0.92]
Trigka 2013	45	71	29	31	4.1%	0.12 [0.03, 0.54]
**Subtotal (95% CI)**		**96**		**70**	**9.0%**	**0.23 [0.09, 0.58]**
Total events	57		58			
Heterogeneity: Tau² = 0.06; Chi² = 1.13, df = 1 (P = 0.29); I² = 12%						
Test for overall effect: Z = 3.11 (P = 0.002)						
**1.2.3 TNM Ⅰ - Ⅱ**						
Zhou 2017-C1	1	34	34	115	3.2%	0.07 [0.01, 0.55]
Zhou 2017-C2	0	28	18	129	2.2%	0.11 [0.01, 1.81]
**Subtotal (95% CI)**		**62**		**244**	**5.5%**	**0.08 [0.02, 0.43]**
Total events	1		52			
Heterogeneity: Tau² = 0.00; Chi² = 0.05, df = 1 (P = 0.83); I² = 0%						
Test for overall effect: Z = 2.97 (P = 0.003)						
**1.2.4 TNM Ⅱ - Ⅳ**						
Chao 2012	4	26	20	34	4.5%	0.13 [0.04, 0.45]
Korkolopoulou 2012	0	61	6	13	2.1%	0.01 [0.00, 0.18]
Setsu 2013	30	59	31	40	5.2%	0.30 [0.12, 0.74]
**Subtotal (95% CI)**		**146**		**87**	**11.8%**	**0.13 [0.03, 0.50]**
Total events	34		57			
Heterogeneity: Tau² = 0.88; Chi² = 5.37, df = 2 (P = 0.07); I² = 63%						
Test for overall effect: Z = 2.94 (P = 0.003)						
**1.2.6 TNM Ⅳ**						
Kasajima 2011-C1	21	24	9	15	4.0%	4.67 [0.95, 22.90]
Kasajima 2011-C2	21	23	10	16	3.7%	6.30 [1.07, 36.94]
Qian 2013	18	25	44	50	4.6%	0.35 [0.10, 1.19]
**Subtotal (95% CI)**		**72**		**81**	**12.2%**	**2.02 [0.29, 14.17]**
Total events	60		63			
Heterogeneity: Tau² = 2.36; Chi² = 9.93, df = 2 (P = 0.007); I² = 80%						
Test for overall effect: Z = 0.71 (P = 0.48)						
**Total (95% CI)**		**1193**		**1204**	**100.0%**	**0.32 [0.18, 0.54]**
Total events	556		687			
Heterogeneity: Tau² = 1.24; Chi² = 113.51, df = 23 (P < 0.00001); I² = 80%						
Test for overall effect: Z = 4.20 (P < 0.0001)						
Test for subgroup differences: Chi² = 7.67, df = 4 (P = 0.10), I² = 47.9%						

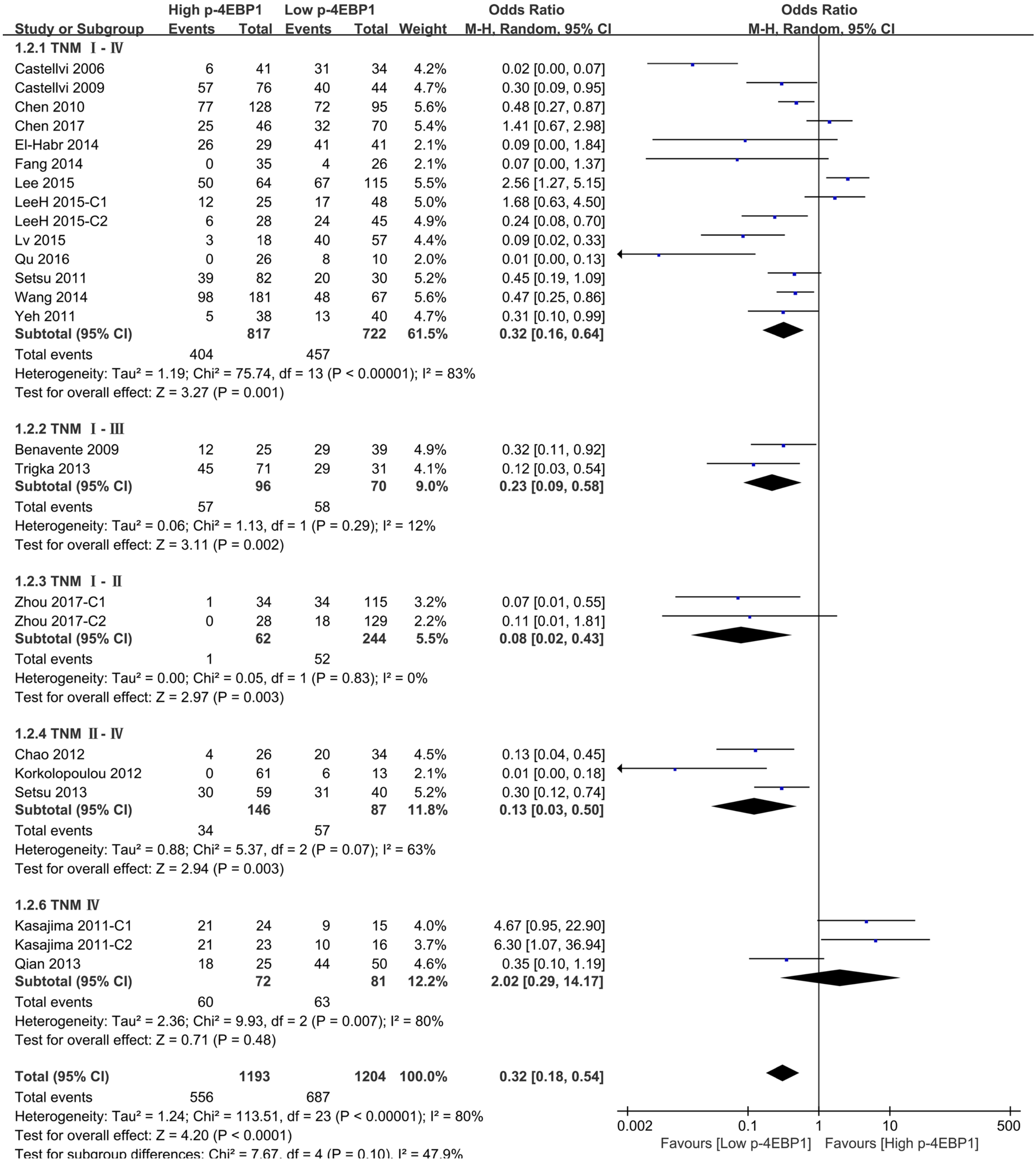

Supplementary Figure 12: The correlation between p-4EBP1 expression and 5-year overall survival based on different TNM stages.

Study or Subgroup	High p-4EBP1 Events	High p-4EBP1 Total	Low p-4EBP1 Events	Low p-4EBP1 Total	Weight	Odds Ratio M-H, Random, 95% CI
**1.1.1 Thr37/46**						
Chao 2012	9	26	21	34	4.5%	0.33 [0.11, 0.95]
Chen 2017	33	46	39	70	5.4%	2.02 [0.91, 4.47]
El-Habr 2014	26	29	41	41	1.3%	0.09 [0.00, 1.84]
Fang 2014	4	35	9	26	3.8%	0.24 [0.07, 0.91]
Florio 2011	10	12	16	19	2.5%	0.94 [0.13, 6.63]
Korkolopoulou 2012	14	61	6	13	4.0%	0.35 [0.10, 1.20]
Lee 2015	50	64	80	115	5.6%	1.56 [0.77, 3.19]
LeeH 2015- Thr37/46	16	25	24	48	4.7%	1.78 [0.66, 4.80]
Malinowsky 2014	29	32	84	86	2.7%	0.23 [0.04, 1.45]
No 2011	66	88	15	15	1.4%	0.10 [0.01, 1.66]
Qian 2013	21	25	48	50	2.8%	0.22 [0.04, 1.29]
Qu 2016	6	26	8	10	2.7%	0.07 [0.01, 0.45]
Setsu 2011	45	82	25	30	4.6%	0.24 [0.08, 0.70]
Setsu 2013	39	59	34	40	4.7%	0.34 [0.12, 0.96]
Trigka 2013	45	71	29	31	3.3%	0.12 [0.03, 0.54]
Wang 2014	112	181	52	67	5.8%	0.47 [0.24, 0.90]
Yeh 2011	9	38	14	40	4.8%	0.58 [0.21, 1.55]
**Subtotal (95% CI)**		**900**		**735**	**64.6%**	**0.44 [0.27, 0.71]**
Total events	534		545			
Heterogeneity: Tau² = 0.58; Chi² = 43.81, df = 16 (P = 0.0002); I² = 63%						
Test for overall effect: Z = 3.35 (P = 0.0008)						
**1.1.2 Thr70**						
Benavente 2009	17	25	35	39	3.8%	0.24 [0.06, 0.92]
Castellvi 2006	24	41	31	34	3.8%	0.14 [0.04, 0.52]
Castellvi 2009	58	76	40	44	4.3%	0.32 [0.10, 1.02]
Cedrés 2012	1	13	2	17	1.7%	0.63 [0.05, 7.75]
Chen 2010	107	128	84	95	5.4%	0.67 [0.30, 1.46]
Kasajima 2011-Cytoplasm	23	24	11	15	2.0%	8.36 [0.83, 83.93]
Kasajima 2011-Nucleus	21	23	13	16	2.5%	2.42 [0.36, 16.50]
LeeH 2015- Thr70	8	28	32	45	4.6%	0.16 [0.06, 0.46]
Wang 2012	2	39	7	38	3.0%	0.24 [0.05, 1.24]
**Subtotal (95% CI)**		**397**		**343**	**31.1%**	**0.42 [0.21, 0.85]**
Total events	261		255			
Heterogeneity: Tau² = 0.59; Chi² = 18.23, df = 8 (P = 0.02); I² = 56%						
Test for overall effect: Z = 2.40 (P = 0.02)						
**1.1.3 Ser65**						
Lv 2015	9	18	45	57	4.4%	0.27 [0.09, 0.82]
**Subtotal (95% CI)**		**18**		**57**	**4.4%**	**0.27 [0.09, 0.82]**
Total events	9		45			
Heterogeneity: Not applicable						
Test for overall effect: Z = 2.31 (P = 0.02)						
**Total (95% CI)**		**1315**		**1135**	**100.0%**	**0.42 [0.29, 0.62]**
Total events	804		845			
Heterogeneity: Tau² = 0.54; Chi² = 65.17, df = 26 (P < 0.0001); I² = 60%						
Test for overall effect: Z = 4.41 (P < 0.0001)						
Test for subgroup differences: Chi² = 0.64, df = 2 (P = 0.73), I² = 0%						

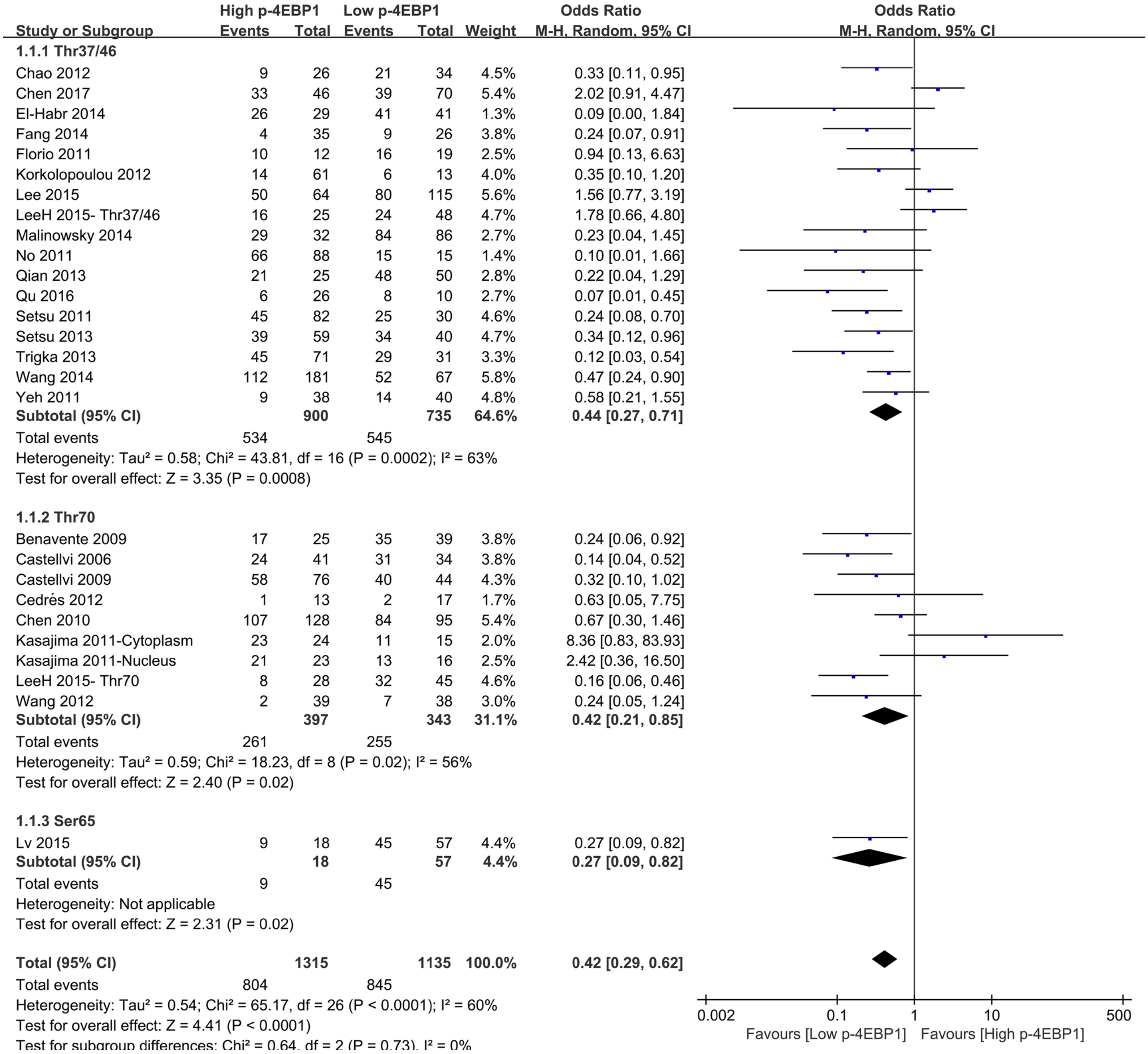

Supplementary Figure 13: The correlation between p-4EBP1 expression and 3-year overall survival based on different phosphorylation sites.

Study or Subgroup	High p-4EBP1 Events	High p-4EBP1 Total	Low p-4EBP1 Events	Low p-4EBP1 Total	Weight	Odds Ratio M-H, Random, 95% CI
**1.2.1 Thr37/46**						
Chao 2012	4	26	20	34	4.4%	0.13 [0.04, 0.45]
Chen 2017	25	46	32	70	5.3%	1.41 [0.67, 2.98]
El-Habr 2014	26	29	41	41	2.0%	0.09 [0.00, 1.84]
Fang 2014	0	35	4	26	2.0%	0.07 [0.00, 1.37]
Florio 2011	7	12	16	19	3.6%	0.26 [0.05, 1.41]
Korkolopoulou 2012	0	61	6	13	2.0%	0.01 [0.00, 0.18]
Lee 2015	50	64	67	115	5.4%	2.56 [1.27, 5.15]
LeeH 2015- Thr37/46	12	25	17	48	4.9%	1.68 [0.63, 4.50]
Malinowsky 2014	27	32	80	86	4.4%	0.41 [0.11, 1.43]
Qian 2013	18	25	44	50	4.5%	0.35 [0.10, 1.19]
Qu 2016	0	26	8	10	1.9%	0.01 [0.00, 0.13]
Setsu 2011	39	82	20	30	5.1%	0.45 [0.19, 1.09]
Setsu 2013	30	59	31	40	5.1%	0.30 [0.12, 0.74]
Trigka 2013	45	71	29	31	4.0%	0.12 [0.03, 0.54]
Wang 2014	98	181	48	67	5.5%	0.47 [0.25, 0.86]
Yeh 2011	5	38	13	40	4.6%	0.31 [0.10, 0.99]
**Subtotal (95% CI)**		**812**		**720**	**64.7%**	**0.35 [0.19, 0.64]**
Total events	386		476			
Heterogeneity: Tau² = 0.99; Chi² = 62.47, df = 15 (P < 0.00001); I² = 76%						
Test for overall effect: Z = 3.42 (P = 0.0006)						
**1.2.2 Thr70**						
Benavente 2009	12	25	29	39	4.8%	0.32 [0.11, 0.92]
Castellvi 2006	6	41	31	34	4.0%	0.02 [0.00, 0.07]
Castellvi 2009	57	76	40	44	4.6%	0.30 [0.09, 0.95]
Chen 2010	77	128	72	95	5.6%	0.48 [0.27, 0.87]
Kasajima 2011-Cytoplasm	21	24	9	15	3.8%	4.67 [0.95, 22.90]
Kasajima 2011-Nucleus	21	23	10	16	3.5%	6.30 [1.07, 36.94]
LeeH 2015- Thr70	6	28	24	45	4.8%	0.24 [0.08, 0.70]
**Subtotal (95% CI)**		**345**		**288**	**31.1%**	**0.44 [0.15, 1.30]**
Total events	200		215			
Heterogeneity: Tau² = 1.71; Chi² = 38.27, df = 6 (P < 0.00001); I² = 84%						
Test for overall effect: Z = 1.49 (P = 0.14)						
**1.2.3 Ser65**						
Lv 2015	3	18	40	57	4.2%	0.09 [0.02, 0.33]
**Subtotal (95% CI)**		**18**		**57**	**4.2%**	**0.08 [0.02, 0.33]**
Total events	3		40			
Heterogeneity: Not applicable						
Test for overall effect: Z = 3.54 (P = 0.0004)						
**Total (95% CI)**		**1175**		**1065**	**100.0%**	**0.34 [0.21, 0.58]**
Total events	589		731			
Heterogeneity: Tau² = 1.14; Chi² = 109.15, df = 23 (P < 0.00001); I² = 79%						
Test for overall effect: Z = 4.07 (P < 0.0001)						
Test for subgroup differences: Chi² = 4.00, df = 2 (P = 0.14), I² = 50.1%						

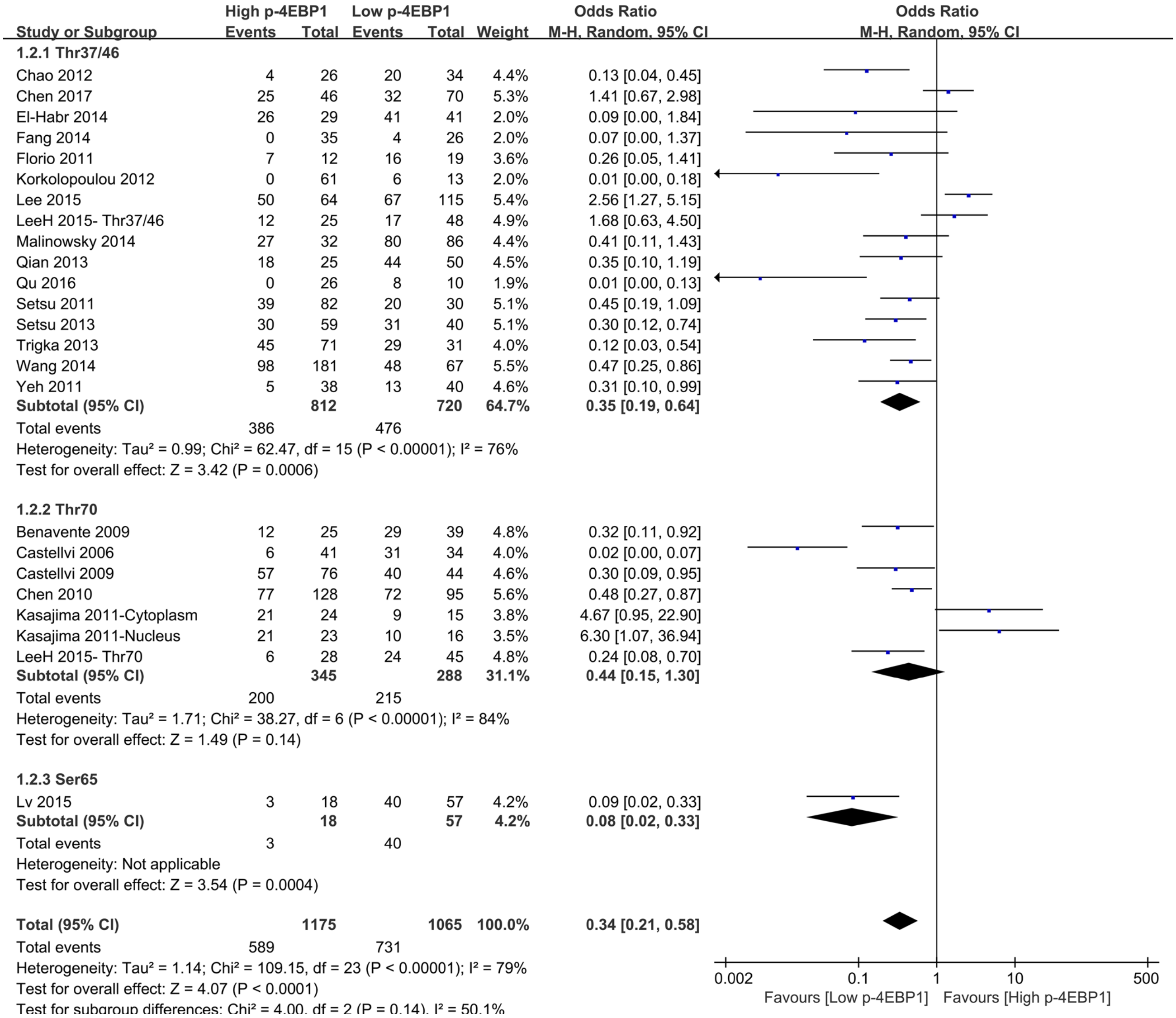

Supplementary Figure 14: The correlation between p-4EBP1 expression and 5-year overall survival based on different phosphorylation sites.

Study or Subgroup	High p-4EBP1 Events	High p-4EBP1 Total	Low p-4EBP1 Events	Low p-4EBP1 Total	Weight	Odds Ratio M-H, Random, 95% CI
**1.1.1 Cytoplasm**						
Benavente 2009	17	25	35	39	3.5%	0.24 [0.06, 0.92]
Cedrés 2012	1	13	2	17	1.6%	0.63 [0.05, 7.75]
Chen 2010	107	128	84	95	5.0%	0.67 [0.30, 1.46]
Kasajima 2011-C1	23	24	11	15	1.8%	8.36 [0.83, 83.93]
No 2011	66	88	15	15	1.3%	0.10 [0.01, 1.66]
Wang 2012	2	39	7	38	2.8%	0.24 [0.05, 1.24]
Wang 2014	112	181	52	67	5.4%	0.47 [0.24, 0.90]
**Subtotal (95% CI)**		**498**		**286**	**21.3%**	**0.49 [0.26, 0.94]**
Total events	328		206			
Heterogeneity: Tau² = 0.24; Chi² = 9.52, df = 6 (P = 0.15); I² = 37%						
Test for overall effect: Z = 2.16 (P = 0.03)						
**1.1.2 Nucleus**						
Castellvi 2006	24	41	31	34	3.5%	0.14 [0.04, 0.52]
Castellvi 2009	58	76	40	44	3.9%	0.32 [0.10, 1.02]
Chen 2017	33	46	39	70	4.9%	2.02 [0.91, 4.47]
Kasajima 2011-C2	21	23	13	16	2.3%	2.42 [0.36, 16.50]
Trigka 2013	45	71	29	31	3.1%	0.12 [0.03, 0.54]
**Subtotal (95% CI)**		**257**		**195**	**17.7%**	**0.48 [0.13, 1.77]**
Total events	181		152			
Heterogeneity: Tau² = 1.75; Chi² = 21.37, df = 4 (P = 0.0003); I² = 81%						
Test for overall effect: Z = 1.10 (P = 0.27)						
**1.1.3 Cytoplasm and nucleus**						
Chao 2012	9	26	21	34	4.2%	0.33 [0.11, 0.95]
El-Habr 2014	26	29	41	41	1.2%	0.09 [0.00, 1.84]
Fang 2014	4	35	9	26	3.5%	0.24 [0.07, 0.91]
Florio 2011	10	12	16	19	2.3%	0.94 [0.13, 6.63]
Korkolopoulou 2012	14	61	6	13	3.7%	0.35 [0.10, 1.20]
Lee 2015	50	64	80	115	5.2%	1.56 [0.77, 3.19]
LeeH 2015-C1	16	25	24	48	4.4%	1.78 [0.66, 4.80]
LeeH 2015-C2	8	28	32	45	4.2%	0.16 [0.06, 0.46]
Lv 2015	9	18	45	57	4.0%	0.27 [0.09, 0.82]
Malinowsky 2014	29	32	84	86	2.4%	0.23 [0.04, 1.45]
Qian 2013	21	25	48	50	2.6%	0.22 [0.04, 1.29]
Qu 2016	6	26	8	10	2.5%	0.07 [0.01, 0.45]
Roh 2015	0	77	9	60	1.3%	0.03 [0.00, 0.61]
Setsu 2011	45	82	25	30	4.2%	0.24 [0.08, 0.70]
Setsu 2013	39	59	34	40	4.3%	0.34 [0.12, 0.96]
Yeh 2011	9	38	14	40	4.4%	0.58 [0.21, 1.55]
Zhou 2017-C1	3	34	34	115	3.7%	0.23 [0.07, 0.81]
Zhou 2017-C2	2	28	21	129	3.1%	0.40 [0.09, 1.79]
**Subtotal (95% CI)**		**699**		**958**	**61.0%**	**0.35 [0.22, 0.55]**
Total events	300		551			
Heterogeneity: Tau² = 0.47; Chi² = 37.40, df = 17 (P = 0.003); I² = 55%						
Test for overall effect: Z = 4.53 (P < 0.00001)						
**Total (95% CI)**		**1454**		**1439**	**100.0%**	**0.40 [0.28, 0.58]**
Total events	809		909			
Heterogeneity: Tau² = 0.53; Chi² = 70.00, df = 29 (P < 0.0001); I² = 59%						
Test for overall effect: Z = 4.92 (P < 0.00001)						
Test for subgroup differences: Chi² = 0.79, df = 2 (P = 0.68), I² = 0%						

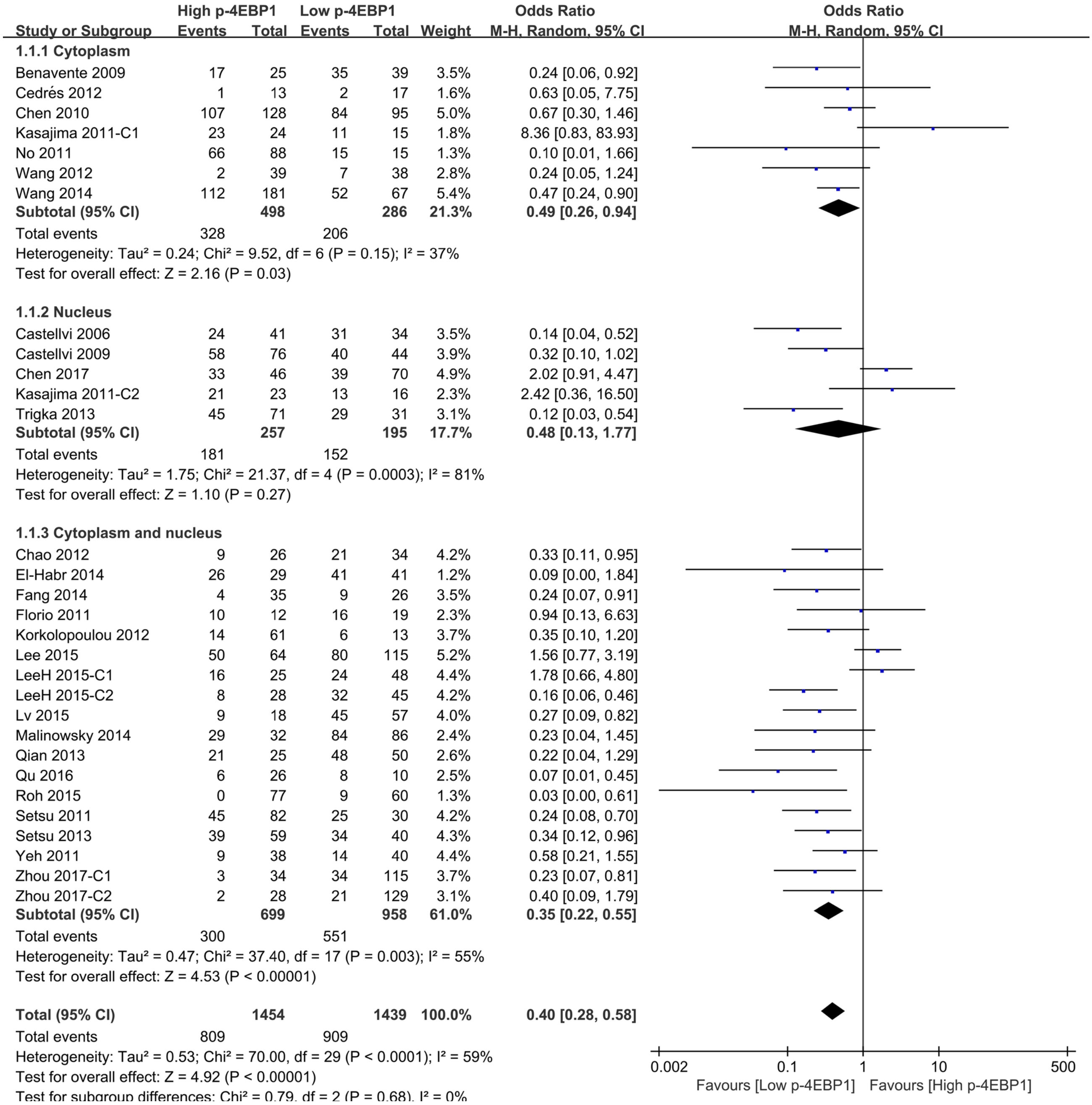

Supplementary Figure 15: The correlation between p-4EBP1 expression and 3-year overall survival based on different subcellular localizations.

Study or Subgroup	High p-4EBP1 Events	High p-4EBP1 Total	Low p-4EBP1 Events	Low p-4EBP1 Total	Weight	Odds Ratio M-H, Random, 95% CI
**1.2.1 Cytoplasm**						
Benavente 2009	12	25	29	39	4.5%	0.32 [0.11, 0.92]
Chen 2010	77	128	72	95	5.3%	0.48 [0.27, 0.87]
Kasajima 2011-C1	21	24	9	15	3.6%	4.67 [0.95, 22.90]
Wang 2014	98	181	48	67	5.3%	0.47 [0.25, 0.86]
**Subtotal (95% CI)**		**358**		**216**	**18.7%**	**0.59 [0.29, 1.20]**
Total events	208		158			
Heterogeneity: Tau² = 0.31; Chi² = 8.32, df = 3 (P = 0.04); I² = 64%						
Test for overall effect: Z = 1.45 (P = 0.15)						
**1.2.2 Nucleus**						
Castellvi 2006	6	41	31	34	3.8%	0.02 [0.00, 0.07]
Castellvi 2009	57	76	40	44	4.4%	0.30 [0.09, 0.95]
Chen 2017	25	46	32	70	5.1%	1.41 [0.67, 2.98]
Kasajima 2011-C2	21	23	10	16	3.3%	6.30 [1.07, 36.94]
Trigka 2013	45	71	29	31	3.8%	0.12 [0.03, 0.54]
**Subtotal (95% CI)**		**257**		**195**	**20.4%**	**0.35 [0.06, 2.07]**
Total events	154		142			
Heterogeneity: Tau² = 3.64; Chi² = 40.78, df = 4 (P < 0.00001); I² = 90%						
Test for overall effect: Z = 1.16 (P = 0.25)						
**1.2.3 Cytoplasm and nucleus**						
Chao 2012	4	26	20	34	4.2%	0.13 [0.04, 0.45]
El-Habr 2014	26	29	41	41	1.9%	0.09 [0.00, 1.84]
Fang 2014	0	35	4	26	1.9%	0.07 [0.00, 1.37]
Florio 2011	7	12	16	19	3.5%	0.26 [0.05, 1.41]
Korkolopoulou 2012	0	61	6	13	1.9%	0.01 [0.00, 0.18]
Lee 2015	50	64	67	115	5.1%	2.56 [1.27, 5.15]
LeeH 2015-C1	12	25	17	48	4.7%	1.68 [0.63, 4.50]
LeeH 2015-C2	6	28	24	45	4.5%	0.24 [0.08, 0.70]
Lv 2015	3	18	40	57	4.0%	0.09 [0.02, 0.33]
Malinowsky 2014	27	32	80	86	4.2%	0.41 [0.11, 1.43]
Qian 2013	18	25	44	50	4.3%	0.35 [0.10, 1.19]
Qu 2016	0	26	8	10	1.8%	0.01 [0.00, 0.13]
Setsu 2011	39	82	20	30	4.9%	0.45 [0.19, 1.09]
Setsu 2013	30	59	31	40	4.8%	0.30 [0.12, 0.74]
Yeh 2011	5	38	13	40	4.4%	0.31 [0.10, 0.99]
Zhou 2017-C1	1	34	34	115	3.0%	0.07 [0.01, 0.55]
Zhou 2017-C2	0	28	18	129	2.0%	0.11 [0.01, 1.81]
**Subtotal (95% CI)**		**622**		**898**	**60.9%**	**0.24 [0.12, 0.47]**
Total events	228		483			
Heterogeneity: Tau² = 1.29; Chi² = 64.61, df = 16 (P < 0.00001); I² = 75%						
Test for overall effect: Z = 4.16 (P < 0.0001)						
**Total (95% CI)**		**1237**		**1309**	**100.0%**	**0.32 [0.19, 0.53]**
Total events	590		783			
Heterogeneity: Tau² = 1.16; Chi² = 113.96, df = 25 (P < 0.00001); I² = 78%						
Test for overall effect: Z = 4.44 (P < 0.00001)						
Test for subgroup differences: Chi² = 3.26, df = 2 (P = 0.20), I² = 38.7%						

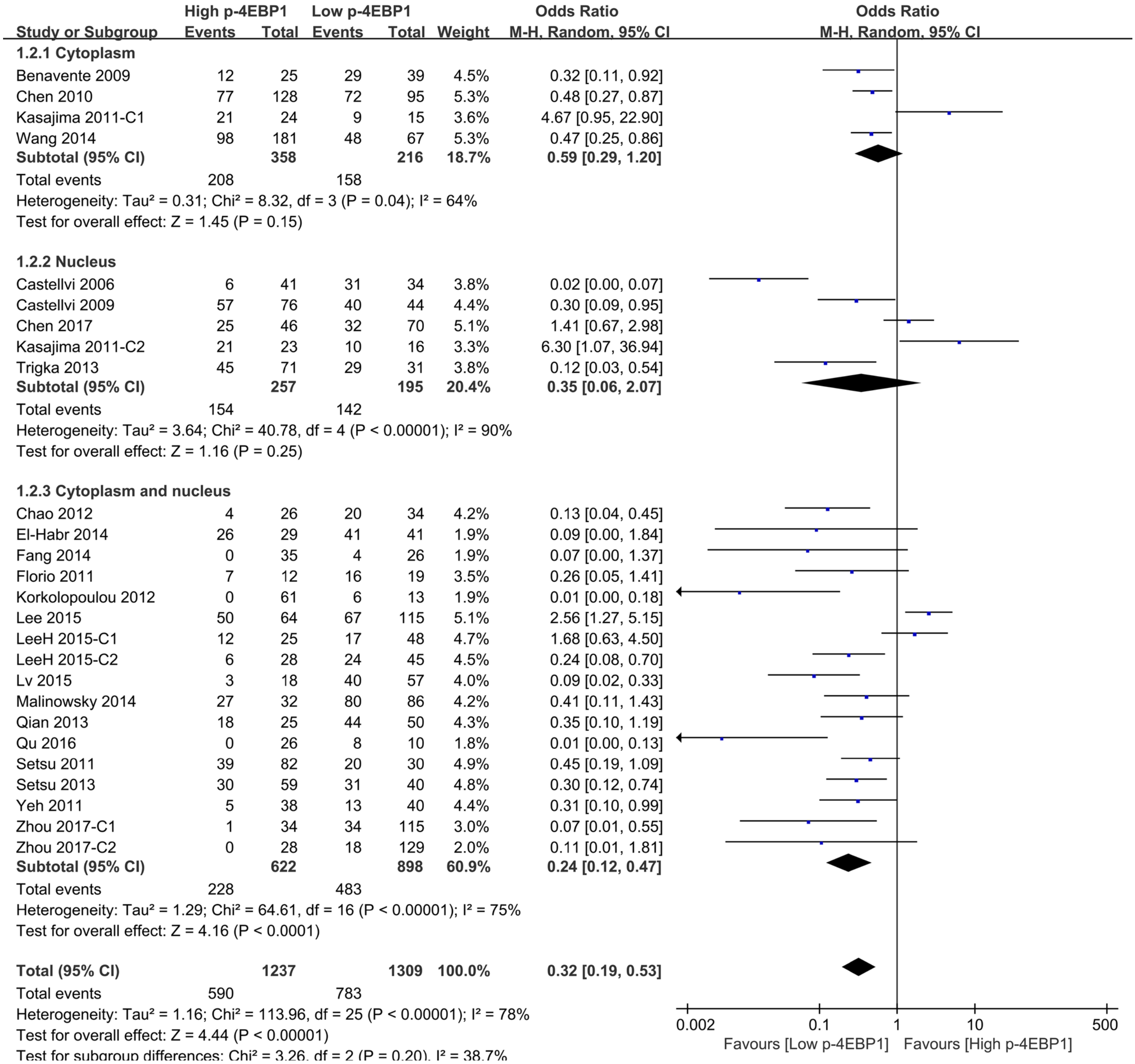

Supplementary Figure 16: The correlation between p-4EBP1 expression and 5-year overall survival based on different subcellular localizations.

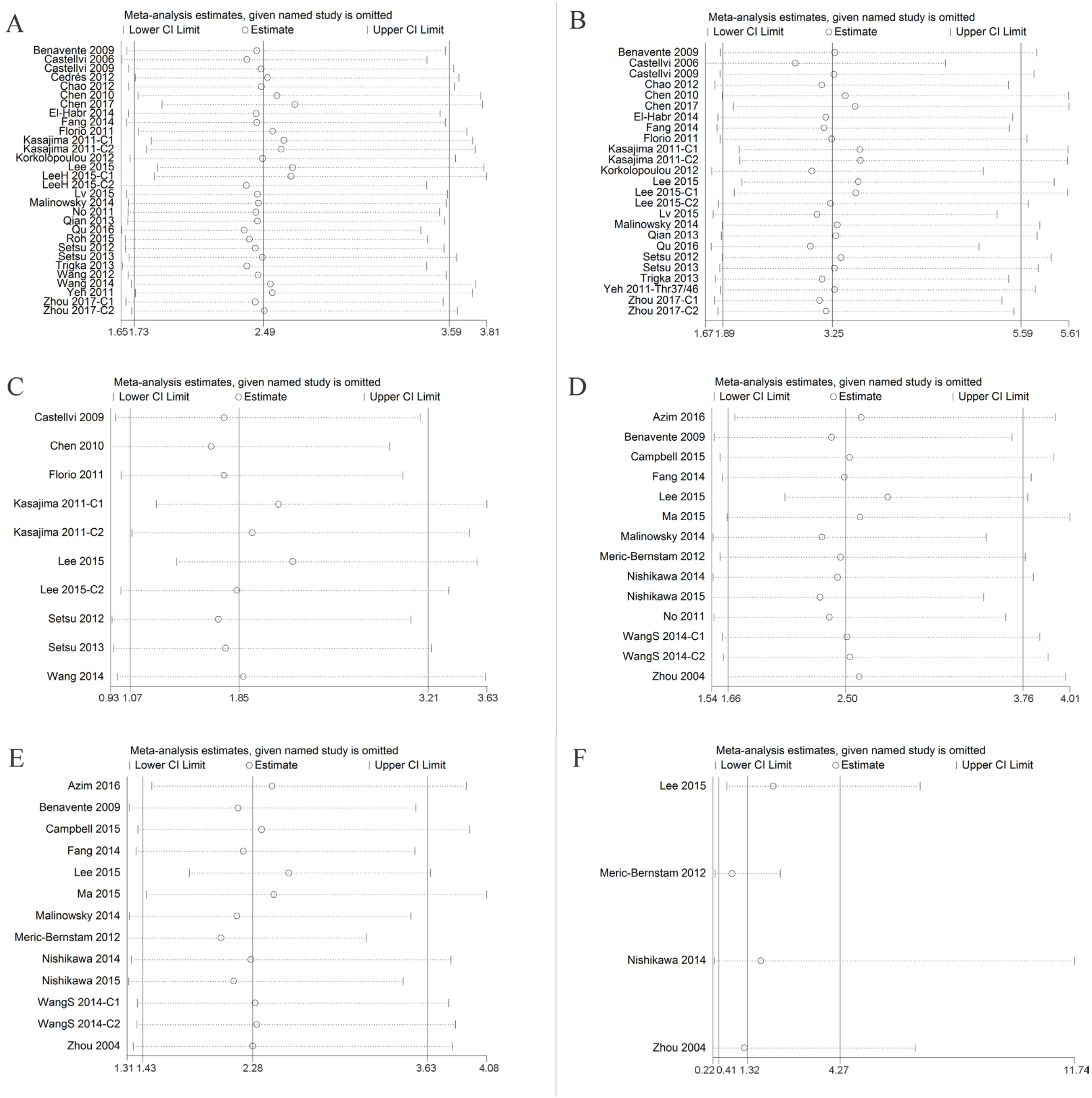

Supplementary Figure 17: Sensitivity analysis of (**A**) 3-year overall survival, (**B**) 5-year overall survival, (**C**) 10-year overall survival, (**D**) 3-year disease-free survival, (**E**) 5-year disease-free survival, and (**F**) 10-year disease-free survival.

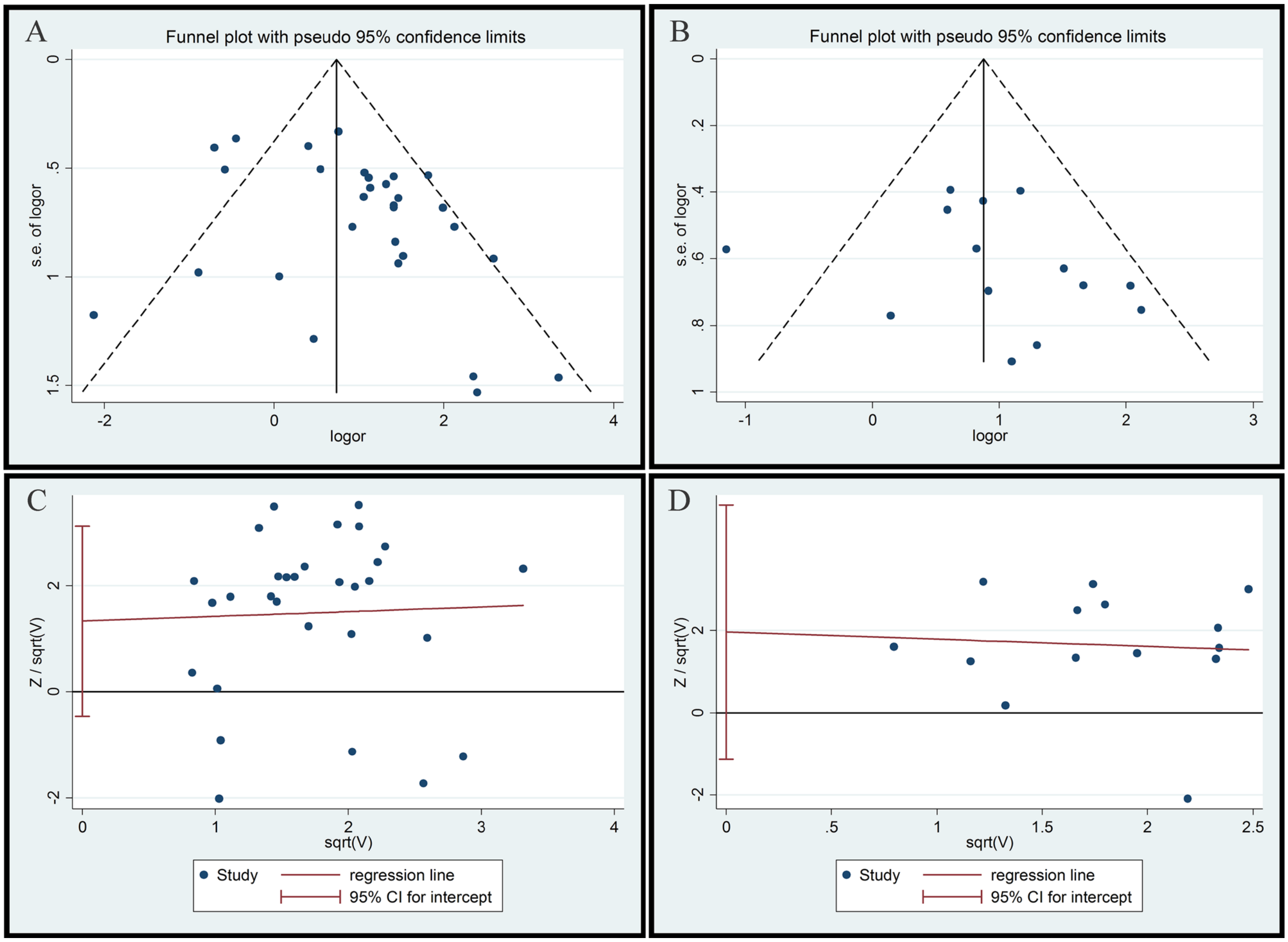

Supplementary Figure 18: The funnel plot of (**A**) 3-year overall survival, (**B**) 3-year disease-free survival, Harbord test for (**C**) 3-year overall survival, and (**D**) 3-year disease-free survival.

Supplementary Table 1: Baseline features of included studies. See Supplementary_Table_1
