# Supplementary material for: Meta-analysis of the prognostic value of p-4EBP1 in human malignancies
Source: Oncotarget. 2017 Dec 7;9(2):2761–9. doi: 10.18632/oncotarget.23031 (PMC5788677; doi:10.18632/oncotarget.23031)
Supplement: Supplementary file 2 [file oncotarget-09-2761-s002.docx]

Supplementary Table 1: Baseline features of included studies

| Study | Country | Tumor  type | TNM stage | Detection  method | p-4EBP1 level | Sample  size | Phosphoryl-ation site | Subcellular  localization | Sex  (M/F) | Mean age(y) | Follow-up months | NOS  score |
| --- | --- | --- | --- | --- | --- | --- | --- | --- | --- | --- | --- | --- |
| Azim 2016 | France | Breast cancer | NA | IHC | Low  High | 14  19 | NA | Nucleus and  cytoplasm | All  female | 51.3±14.25 | 56.4(25.5-82.8) | 6 |
| Benavente 2009 | Spain | Cervical cancer | Ⅰ-Ⅲ | IHC | Low  High | 39  25 | Thr70 | Cytoplasm | All  female | 57.8 ±14.8 | 40 | 6 |
| Campbell 2015 | UK | Renal cancer | Ⅰ-Ⅳ | IHC | Low  High | 70  43 | Ser65 | Nucleus and  cytoplasm | NA | 63±8.83 | NA | 8 |
| Castellvi 2006 | Spain | Ovarian Cancer | Ⅰ-Ⅳ | IHC | Low  High | 34  41 | Thr70 | Nucleus | All  female | 55±11.17 | 31(24-80) | 6 |
| Castellvi 2009 | Spain | Endometrial cancer | Ⅰ-Ⅳ | IHC | Low  High | 44  76 | Thr70 | Nucleus | All  female | 63±7 | 63 | 6 |
| Cedrés 2012 | Spain | Malignant pleural mesothelioma | Ⅲ-Ⅳ | IHC | Low  High | 17  13 | Thr70 | Cytoplasm | 25/5 | 68±7.75 | 8.8 | 6 |
| Chao 2012 | China | Esophageal cancer | Ⅱ-Ⅳ | IHC | Low  High | 34  26 | Thr37/46 | Nucleus and  cytoplasm | 58/2 | 56±11.77 | 55(28-71) | 8 |
| Chen 2010 | China | Nasopharyngeal cancer | Ⅰ-Ⅳ | IHC | Low  High | 95  128 | Thr70 | Cytoplasm | NA | 46±12 | NA | 6 |
| Chen 2017 | China | Colorectal cancer | Ⅰ-Ⅳ | IHC | Low  High | 70  46 | Thr37/46 | Nucleus | 80/36 | NA | NA | 7 |
| El-Habr 2014 | Greece | Meningioma | Ⅰ-Ⅳ | IHC | Low  High | 41  29 | Thr37/46 | Nucleus and  cytoplasm | 27/44 | 62±11 | 74.7(0.7-146.3) | 7 |
| Fang 2014 | China | Cholangiocarcinom-a | Ⅰ-Ⅳ | IHC | Low  High | 26  35 | Thr37/46 | Nucleus and  cytoplasm | 45/16 | 55 | 74.7(0.7-146.3) | 8 |
| Florio 2011 | Italy | Pancreatic cancer | NA | IHC | Low  High | 19  12 | Thr37/46 | Nucleus and  cytoplasm | NA | NA | NA | **4** |
| Kasajima 2011  -C1 | Germany | GEP-NET | Ⅳ | IHC | Low  High | 15  24 | Thr70 | Cytoplasm | NA | 58.2 | 48.3(3.5-210.7) | 8 |
| Kasajima 2011  -C2 | Germany | GEP-NET | Ⅳ | IHC | Low  High | 16  23 | Thr70 | Nucleus | NA | 58.2 | 48.3(3.5-210.7) | 8 |
| Korkolopoulou 2012 | Greece | Astrocytomas | Ⅱ-Ⅳ | IHC | Low  High | 13  61 | Thr37/46 | Nucleus and  cytoplasm | NA | 58±10.83 | NA | 6 |
| Lee 2015 | South Korea | Gastric cancer | Ⅰ-Ⅳ | IHC | Low  High | 115  64 | Thr37/46 | Nucleus and  cytoplasm | 118/61 | 63±7.67 | 96(1-139) | 7 |
| LeeH 2015-C1 | South Korea | Lung cancer | Ⅰ-Ⅳ | IHC | Low  High | 48  25 | Thr37/46 | Nucleus and  cytoplasm | 59/14 | 64±8.5 | 30(1-135) | 8 |
| LeeH 2015-C2 | South Korea | Lung cancer | Ⅰ-Ⅳ | IHC | Low  High | 45  28 | Thr70 | Nucleus and  cytoplasm | 59/14 | 64±8.5 | 30(1-135) | 8 |
| Lv 2015 | China | Lung cancer | Ⅰ-Ⅳ | IHC | Low  High | 57  18 | Ser65 | Nucleus and  cytoplasm | 39/36 | 58.8 ± 11.9 | NA | 8 |
| Ma 2015 | China | Breast cancer | Ⅰ-Ⅳ | IHC | Low  High | 211  74 | Thr70 | Nucleus and  cytoplasm | All  female | NA | 71.8 | 8 |
| Malinowsky 2014 | Germany | Colorectal cancer | Ⅱ | RPPA | Low(DFS/OS)  High(DFS/OS) | 78/86  24/32 | Thr37/46 | Nucleus and  cytoplasm | NA | 65±9.83 | 97.2 | 7 |
| Meric-Bernstam 2012 | USA | Breast cancer | Ⅰ-Ⅲ | RPPA | Low  High | 180  8 | Ser65 | Nucleus and  cytoplasm | All  female | 68±9.83 | 87(1-197) | 7 |
| Nishikawa 2014 | Japan | Renal cancer | Ⅰ-Ⅲ | IHC | Low  High | 97  40 | NA | Nucleus and  cytoplasm | 99/38 | 65.0±8.67 | 63.5(7-181) | 7 |
| Nishikawa 2015 | Japan | Bladder cancer | Ⅰ-Ⅳ | IHC | Low  High | 28  21 | NA | Nucleus and  cytoplasm | 39/10 | 74±10.5 | NA | 7 |
| No 2011 | South Korea | Ovarian cancer | Ⅰ-Ⅳ | IHC | Low  High | 88  15 | Thr37/46 | Cytoplasm | All  female | 50±10.67 | 49(4-82) | 7 |
| Qian 2013 | USA | Neuroendocrine cancer | Ⅳ | IHC | Low  High | 50  25 | Thr37/46 | Nucleus and  cytoplasm | NA | 54.8±13 | 61.2 | 6 |
| Qu 2016 | China | Renal cancer | Ⅰ-Ⅳ | IHC | Low  High | 10  26 | Thr37/46 | Nucleus and  cytoplasm | 13/23 | 29±12.25 | 30(2-87) | 8 |
| Roh 2015 | South Korea | Lung cancer | Ⅰ-Ⅳ | IHC | Low  High | 77  40 | NA | Nucleus and  cytoplasm | 89/28 | 68±8.33 | 2.3(0.1-110) | 8 |
| Setsu 2012 | Japan | Leiomyosarcomas | Ⅰ-Ⅳ | IHC | Low  High | 30  82 | Thr37/46 | Nucleus and  cytoplasm | NA | 60±12.33 | 27(1-346) | 6 |
| Setsu 2013 | Japan | Synovial sarcoma | Ⅱ-Ⅳ | IHC | Low  High | 40  59 | Thr37/46 | Nucleus and  cytoplasm | NA | NA | 51(1-278) | 8 |
| Trigka 2013 | Greece | Lung cancer | Ⅰ-Ⅲ | IHC | Low  High | 31  71 | Thr37/46 | Nucleus | 84/14 | 71±1.17 | 33(2-108) | 6 |
| Wang 2012 | China | Cholangiocarcinom-a | Ⅰ-Ⅲ | IHC | Low  High | 38  39 | Thr70 | Cytoplasm | 53/24 | NA | NA | 7 |
| Wang 2014 | China | Nasopharyngeal cancer | Ⅰ-Ⅳ | IHC | Low  High | 67  181 | Thr37/46 | Cytoplasm | 187/61 | 46.8±9.33 | 44.6(10-120) | 8 |
| WangS 2014-C1 | China | Breast cancer | Ⅱ-Ⅲ | IHC | Low  High | 14  69 | Thr36 | Nucleus and  cytoplasm | All female | NA | 45 (32-78) | 6 |
| WangS 2014-C2 | China | Breast cancer | Ⅰ-Ⅲ | IHC | Low  High | 25  58 | Thr36 | Nucleus and  cytoplasm | All female | NA | 45 (32-78) | 6 |
| Yeh 2011 | China | Esophageal cancer | Ⅰ-Ⅳ | IHC | Low  High | 40  38 | Thr37/46 | Nucleus and  cytoplasm | 75/3 | 57±12 | NA | 8 |
| Zhou 2004 | China | Breast cancer | Ⅰ-Ⅲ | IHC | Low  High | 47  70 | Ser65 | Nucleus and  cytoplasm | All female | NA | 76.4 | 8 |
| Zhou 2017-C1 | China | Pancreatic cancer | Ⅰ-Ⅱ | IHC | Low  High | 115  34 | NA | Nucleus and  cytoplasm | 97/52 | 63±7.67 | 12.7(1-87) | 6 |
| Zhou 2017-C2 | China | Pancreatic cancer | Ⅰ-Ⅱ | IHC | Low  High | 129  28 | NA | Nucleus and  cytoplasm | 89/68 | 65±9 | 11.5(2-95) | 6 |

M/F: male/female; NOS: Newcastle-Ottawa Scale; IHC: immunohistochemistry; NA: not available; RPPA: reverse-phase protein arrays; DFS: disease free survival; OS: overall survival; GEP-NET: gastroenteropancreatic neuroendocrine tumors; Ser: serine; Thr: threonine; C 1: cohort 1; C2: cohort 2.
